# *In situ* XAFS–XRD study of the Zr–Y_2_O_3_ interaction at extra-high temperatures

**DOI:** 10.1107/S1600577524003321

**Published:** 2024-05-31

**Authors:** Ayumi Itoh, Satoru Matsuo, Kenta Yoshida, Kenji Konashi, Rikuto Ikuta, Keisuke Niino, Yuji Arita, Masaaki Kobata, Tatsuo Fukuda, Tohru Kobayashi, Hajime Tanida, Tsuyoshi Yaita

**Affiliations:** a Laboratory for Zero-Carbon Energy Tokyo Institute of Technology 2-12-1 N1-3Ookayama, Meguro-ku Tokyo152-8550 Japan; b Institute of Materials Research Tohoku University 2145-2Narita-cho, Oarai-cho, Ibaraki-gun Ibaraki311-1313 Japan; c Research Institute of Nuclear Engineering University of Fukui 1-3-33Tetsuwa-cho, Tsuruga-shi Fukui914-0055 Japan; d Materials Science Research Center Japan Atomic Energy Agency 1-1-1Kouto, Sayo-cho, Sayo-gun Hyogo679-5148 Japan; RIKEN SPring-8 Center, Japan

**Keywords:** *in situ* XAFS–XRD, Zr–Y_2_O_3_, extra-high temperature., Zr–Y2O3

## Abstract

The *in situ* measurement technique for a metal/metal-oxide mixture at extra-high temperature above 2000 K has been desired in the field of nuclear safety engineering. In the present study, we succeeded in simultaneous XAFS–XRD measurements of the Zr oxidation [Zr + O → Zr(O) + ZrO_2_] up to 1952 K and ZrO_2_–Y_2_O_3_ reaction from 1952 to 2519 K. The chemical shift during Zr oxidation was observed in the absorption spectra around the Zr *K*-edge, and the interatomic cation–cation and cation–oxygen distances obtained by the fitting analysis of EXAFS during the Y_2_O_3_–ZrO_2_ reaction are explained. Also, the temperature dependency of the anharmonic effect was investigated by comparing the fitted second- and third-order cumulants with the theoretical ones in which the Morse potential was applied as an interatomic potential, giving a good explanation about the local structure dynamics. Finally, the applicability of the developed system to investigation of nuclear fuel materials, such as UO_2_–Zr, is discussed.

## Abstract 2

*In situ* XAFS–XRD measurements of Zr oxidation and the ZrO_2_–Y_2_O_3_ reaction up to 2519 K are reported. Analysis of anharmonic effects at high temperatures showed that Y_2_O_3_–ZrO_2_ proceeded to stabilize Zr—O and Y—O bonding. The developed system was confirmed to be applicable for high-temperature investigations of nuclear fuel material.

## 1. Introduction

For the safety evaluation of nuclear fuel materials, it is important to obtain knowledge on the high-temperature reactions that cause fuel-cladding melting. In previous fuel-melting experiments, UO_2_–Zr diffusion couples were heated to a target temperature using an induction furnace, held for a certain period of time, cooled to room temperature, and then the samples were chemically analyzed to evaluate the extent of the reaction. According to the results, it has been reported that the melting amount of UO_2_ in Zr rapidly proceeds above approximately 2273 K (Hofmann & Politis, 1979 ▸; Tim & Olander, 1988 ▸; Kim & Olander, 1988 ▸; Hayward & George, 1994 ▸, 1996 ▸; Hayward *et al.*, 1999 ▸; Shirasu *et al.*, 2023 ▸). However, the analytical results obtained by such a method include large uncertainties derived from reactions during the heating and cooling processes, and thus it is difficult to estimate the precise chemical conditions in which the melting reaction begins. To elucidate the state of the materials and the intrinsic physicochemical state at high temperature, *in situ* observation techniques using synchrotron radiation have attracted attention.

X-ray absorption fine structure (XAFS) is a technique for investigating the local atomic structure in both solid and liquid phases and has been successfully applied to many types and states of materials. Recently, XAFS has been applied to nuclear materials, such as molten rare-earth oxides (Okamoto *et al.*, 2017 ▸) and molten salts at high temperatures (Bessada *et al.*, 2017 ▸, 2020 ▸). *In situ*X-ray diffraction (XRD) measurement is also a powerful technique for tracing structural transitions during heat treatment and has been applied to molten UO_2_ coupled with the laser ablation method (Skinner *et al.*, 2014 ▸). These two techniques complement each other, and coupling XAFS with XRD has often been applied for investigation of the reaction kinetics of functional materials (such as catalysts). However, few studies have been reported on the application of the combined XAFS–XRD technique at high temperature.

In the present study, we focus on the *in situ* XAFS–XRD technique to investigate the high-temperature interaction (above 2000 K) of Zr–Y_2_O_3_ mixture by additionally installing XRD apparatus to the XAFS measurement system developed by one of authors (Niino *et al.*, 2023 ▸). Firstly, the temperature of the sample was calibrated using a heating test with the Y_2_O_3_ powder from room temperature to approximately 2700 K. Thereafter, a specimen of Zr–Y_2_O_3_ (Zr:Y_2_O_3_ = 50:50 wt%) was prepared and then heated up to approximately 2500 K. The X-ray absorption and diffraction images from the transmitted X-rays were recorded, which enabled phase relations and the local structures around metals (Y and Zr) to be studied. Our results are compared with the literature regarding the Y_2_O_3_ and ZrO_2_–Y_2_O_3_ system to check the validity of the improved system, and then its applicability to the Zr–UO_2_ interaction in the future is discussed.

## 2. Experimental procedure

### 2.1. Measurement system

**Figure 1:**
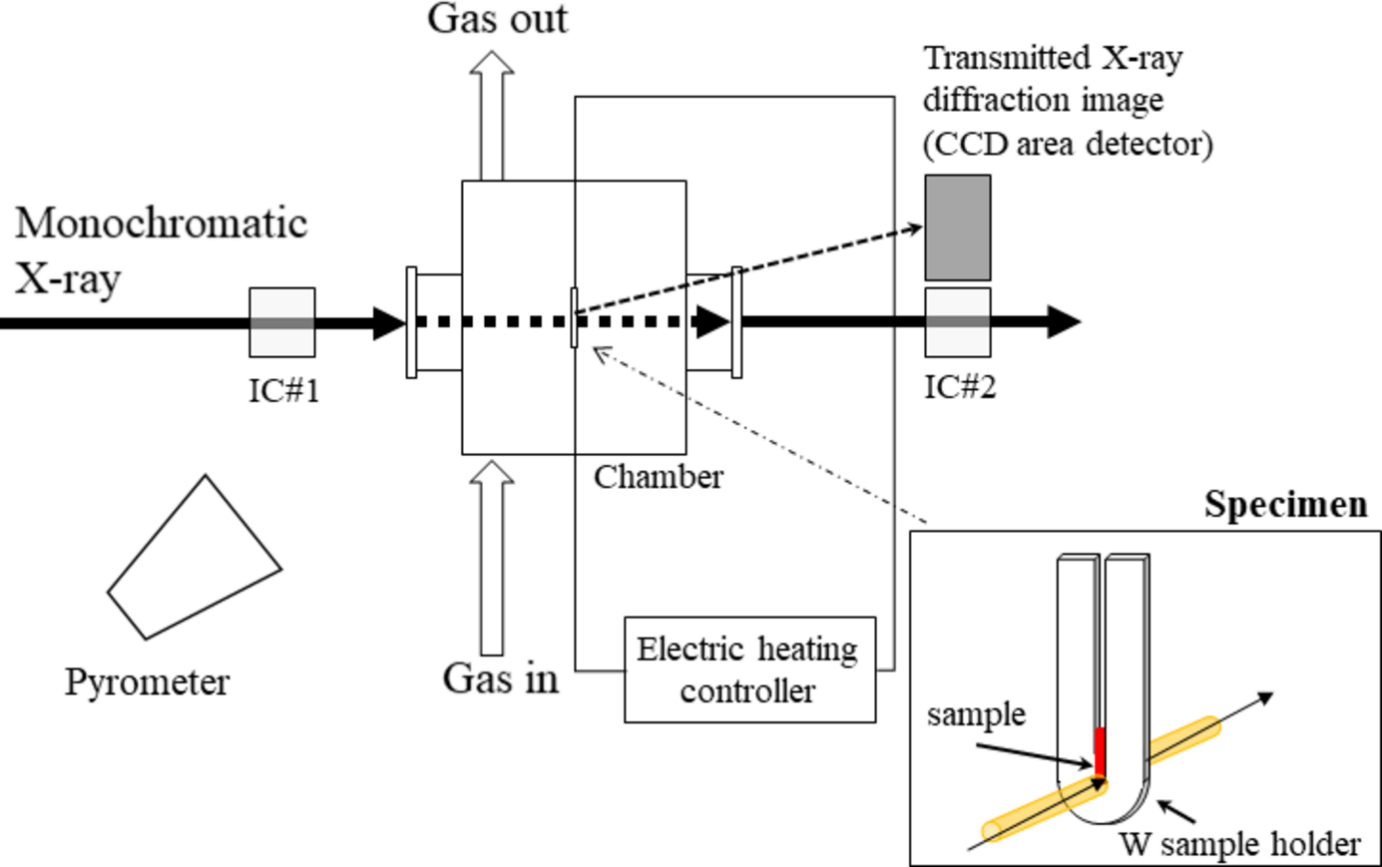
Schematic diagram of the XAFS/XRD measurement apparatus and heating system.

Fig. 1 ▸ shows a schematic diagram of the XAFS–XRD measurement system. We installed the heating chamber and collected the XAFS and XRD data at beamline BL22XU (Shobu *et al.*, 2007 ▸) at SPring-8 (Hyogo, Japan), which can generate X-rays with energies in the range 5–70 keV. A monochromator crystal of Si(111), equipped in a cam-type double-crystal monochromator (Kohzu Precision Co. Ltd) (Yabashi *et al.*, 1999 ▸), was utilized for obtaining the X-rays for measurement. In this study, X-rays with energies in the range 16.839–18.561 keV were used. A nickel-coated mirror (bent cylindrical mirror with glancing angle of 1.9 mrad) installed downstream of the monochromator focused the X-rays and rejected higher harmonics, and another nickel-coated flat mirror returned the beam direction to horizontal. In the measurement, at first, X-rays with an energy of 17.72 keV transmitted through the sample were captured by a 1024 × 1024 pixel CCD area detector (Hamamatsu Photonics KK) to obtain the diffraction image. For XAFS in transmission, the monochromator was quick-scanned between 16.839 and 18.561 keV to cover both the Y *K*-edge (17.038 keV) and the Zr *K*-edge (17.998 keV). The data collection time was 20 s for XAFS and 10 s for the diffraction image at each temperature. The X-ray beams were focused to an area of approximately 0.4 mm^2^ and the XAFS measurements were performed in the transmission mode using two ionization chambers (IC#1, IC#2) filled with nitro­gen gas to count the photons absorbed by the sample. The specimens with sample material (prepared as explained in the next section) were placed in a stainless-steel heating chamber. The chamber had two quartz-glass windows of thickness 6 mm and diameter 60 mm for pyrometer use. Each glass window had a hole at the center and the hole was covered with polyimide film (0.069 mm in thickness). The X-ray beam passed through the polyimide film. Sample material was placed in the slit of a tungsten sample holder. Electrodes attached to the sample holder were connected to a programmable DC power supply (TAKASAGO Ltd) (0–6 V, 0–400 A) to enable effective heating of the sample materials. The temperature of the sample was monitored by a one-color pyrometer (LUMASENSE IGA 740-LO). The temperature measuring spot was 1.5 mm at a distance of 300 mm, which covered the tungsten holder around the sample. The temperature reached using the pyrometer was corrected by an W-Re type thermocouple; however, uncertainty inevitably arises above approximately 2273 K. Therefore, the temperature was corrected with the lattice parameter of Y_2_O_3_ as explained in Section 3.1. At the beginning of the measurement, the chamber was filled with pure argon gas (99.9999% purity) and vacuumed at ∼10^4^ Pa. Details of the sample holder are given by Niino *et al.* (2023 ▸) and shown in Fig. S1 of the supporting information.

### 2.2. Sample preparation

Specimens were prepared by mixing Y_2_O_3_ powder (99.99% purity, Kojundo Chemical Laboratory Co. Ltd) and iso­propyl alcohol (extra-pure reagent, Kanto CHEMICAL Co. Inc.) in a mortar to produce a slurry. To test the Zr–Y_2_O_3_ mixture (Zr:Y_2_O_3_ = 50:50 wt%), Zr powder (99.9% purity, FUJIFILM Wako Pure Chemical Co. Ltd) was mixed with Y_2_O_3_ powder. The slurry sample was pasted onto the slit (60 µm wide) of the W holder, dried, and stabilized by heating at 673 K for 20 h. All preparations were conducted in an inert glove box. After the XAFS–XRD measurements, the specimens were removed from the heating chamber. Then metallographic analyses were performed via scanning electron microscopy with electron dispersion spectroscopy (SEM/EDS; JSM-6010 LV, Jeol) and transmission electron microscopy with a spherical aberration corrector (AC-TEM; ARM 200F, Jeol) to investigate the final state of the sample materials.

### 2.3. Data analysis

The diffraction images were transformed into intensity profiles using *IPAnalyzer* software (Seto *et al.*, 2010 ▸). Diffraction patterns from the irradiated X-ray energy in this study (17.72 keV) were calculated using *RIETAN* software (Kim & Izumi, 1994 ▸), and the peaks were compared with the experimental ones of Swamy *et al.* (1999 ▸).

The XAFS spectra at the Y and Zr *K*-edges were analyzed using the *Athena* software to extract the EXAFS function (Ravel & Newville, 2005 ▸), χ(*k*), expressed as
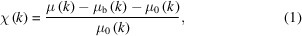

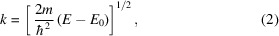
where *k* is the photoelectron wavenumber, ℏ (= *h*/2π) is the reduced Plank constant, *m* is the mass of an electron, *E* is the energy of the incident X-rays, *E*_0_ is the point at which the second-order derivative with respect to energy is zero, μ(*k*) is the total absorption coefficient, μ_b_(*k*) is the background contribution from the excitations of other shells and atoms in the sample, and μ_0_(*k*) is calculated by a least-square fitting with the Victoreen’s formula *aE*^−3^ − *bE*^−4^ − *c* (where *a*, *b* and *c* are constants). The Hamming window function was multiplied by χ(*k*) to avoid the truncation error in the Fourier transformed spectra in *R*-space. The *k*-range for the Fourier transform was from 3 to 10. We obtained the radial structural function (RSF) from the *k*^3^-weighted Fourier transformed magnitude |FT[*k*^3^χ(*k*)]| with the EXAFS function, which were further used to obtain parameters of local structure (coordination number, interatomic distance, Debye–Waller factor and third-order cumulant) by curve fitting analysis with the *Artemis* software (Ravel & Newville, 2005 ▸) with the following formula,
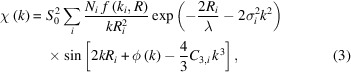
where *R*_*i*_ is the interatomic distance between absorbing and photoelectron scattering atom *i*, *N*_*i*_ is the coordination number, *f*(*k*, *R*_*i*_) and ϕ(*k*) are the backscattering amplitude and total phase shift functions [which were calculated by *FEFF6* code (Rehr & Ankudinov, 2001 ▸) embedded in the *Artemis* software], λ is the photoelectron mean free path, 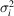 is the second-order cumulant (arising mainly from the harmonic part in the interatomic potential), and *C*_3,*i*_ is the third-order cumulant (giving the anharmonic effect in the interatomic potential). It should be noted that the fourth-order cumulant is also essential in the EXAFS analysis at ultra-high temperature, because it may affect the amplitude of χ(*k*) reflecting the anharmonic effect. However, it was difficult to obtain good fitting results with the EXAFS spectra in this study due to the insufficient signal-to-noise (S/N) ratio at high *k*-range. Therefore, we performed the analysis only up to the third-order cumulant. The 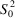 value was firstly determined from the spectrum taken at 300 K (before heating) and was used as fixed values for other temperatures’ fitting analysis.

## 3. Results

### 3.1. Temperature calibration with Y_2_O_3_ measurements

**Figure 2:**
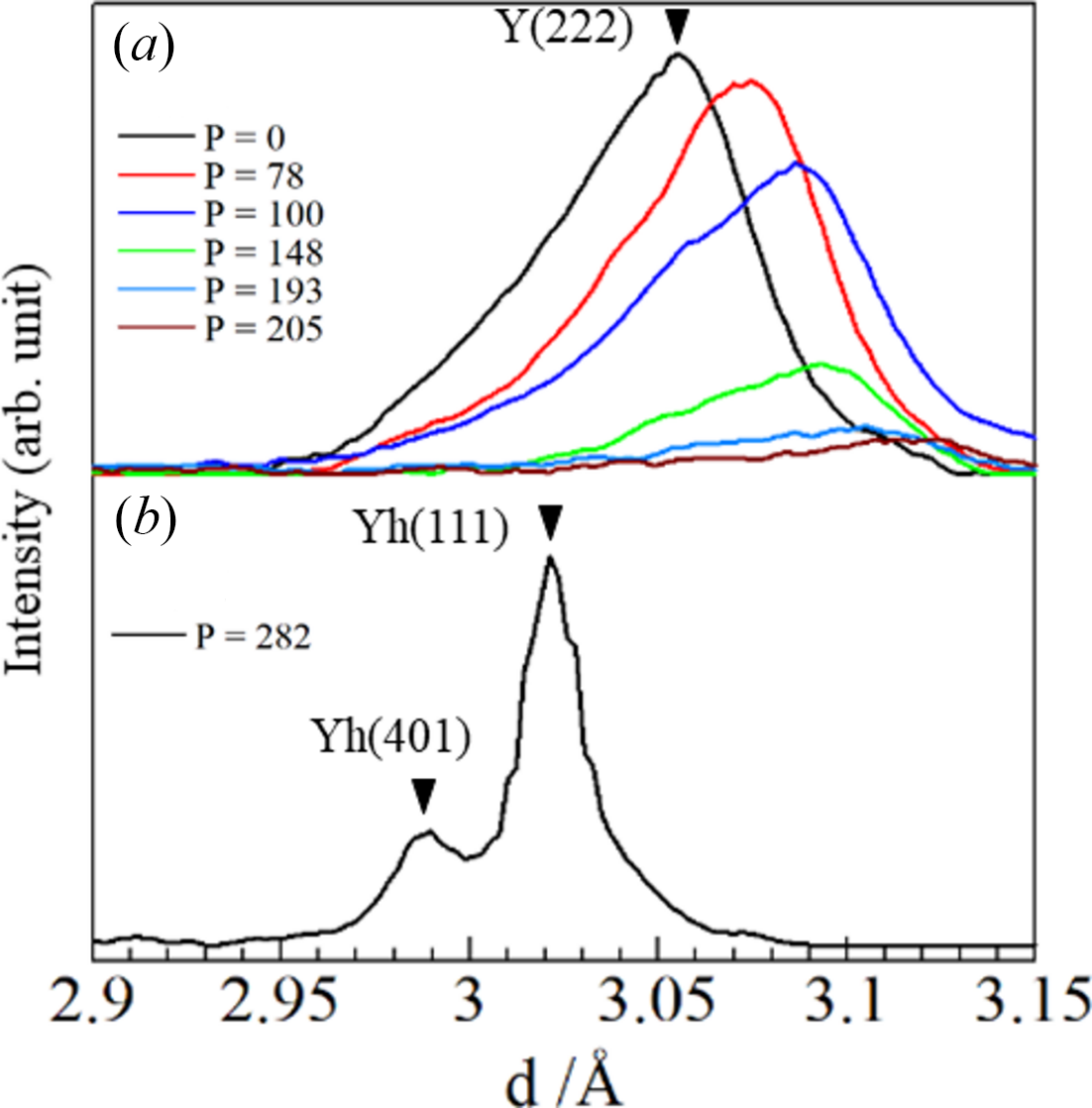
X-ray diffraction spectra obtained at powers of (*a*) 0, 78, 100, 148, 193 and 205 W and (*b*) 282 W. The peak marked as Y(*hkl*) belongs to cubic yttria and Yh(*hkl*) to the high-temperature structure of Y_2_O_3_.

The X-ray patterns obtained at seven different powers (0, 78, 100, 148, 193, 205 and 282 W) are shown in Fig. 2 ▸. The peak labeled Y(*hkl*) represents cubic Y_2_O_3_ in the space group 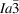 and Yh(*hkl*) represents monoclinic Y_2_O_3_ in the space group *C*2/*m*, which was formed in the temperature range 2500–2600 K (Jacobson *et al.*, 2004 ▸). The diffraction spectrum at 300 K (*P* = 0) exhibited a Debye ring at 2θ = 13.09°, which was identical to that of cubic Y_2_O_3_ calculated using the *RIETAN* code with the crystallographic information file (CIF) (Andrievskaya *et al.*, 1996 ▸) obtained via *AtomWork* (https://crystdb.nims.go.jp/) (Xu *et al.*, 2011 ▸). The corresponding *d*-spacing and unit-cell parameter were calculated as 3.06 and 10.6031 Å, respectively. The peak of the *d*-spacing at high temperature shifted to 3.13 Å (Table 1 ▸). Above *P* = 200 W, the Debye ring disappeared, and spots appeared at *P* = 282 W [Fig. 2 ▸(*b*)]. 2θ of these peaks corresponds to the Bragg diffraction of monoclinic Y_2_O_3_ (Skrikanth *et al.*, 1994 ▸). Immediately after that spot was observed, it disappeared from the diffraction image. At the same time, attenuation in the X-ray absorption was also observed. Based on this observation, the sample was considered to have been in a coarse-grained state at the temperature of the phase transformation from the cubic structure (∼2530 K) to the melting point (2712 K). We note that the diffraction peak width clearly changed at the phase transition temperature; the broad peak in the low-temperature phase [Fig. 2(*a*)] shrinked to almost the resolution limit in the high temperature phase [Fig. 2(*b*)]. The reason for this is not clear, but the sample crystal could be strained during the sample preparation.

**Figure 3:**
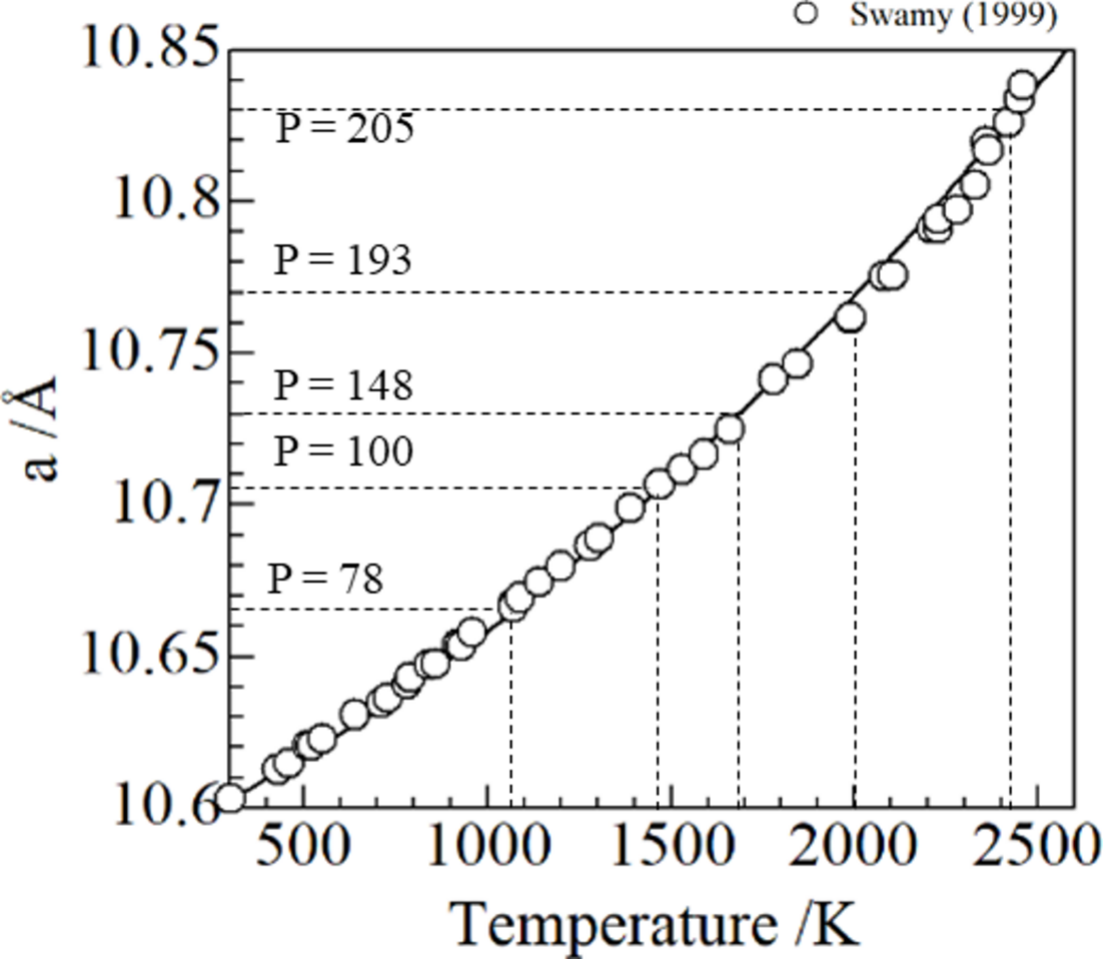
Unit-cell parameter *a* as a function of temperature obtained by Swamy *et al.* (1999 ▸) with a fitting curve (solid line). The lattice parameters obtained in this study at 78, 100, 193, 205 and 282 W are indicated together.

**Figure 4:**
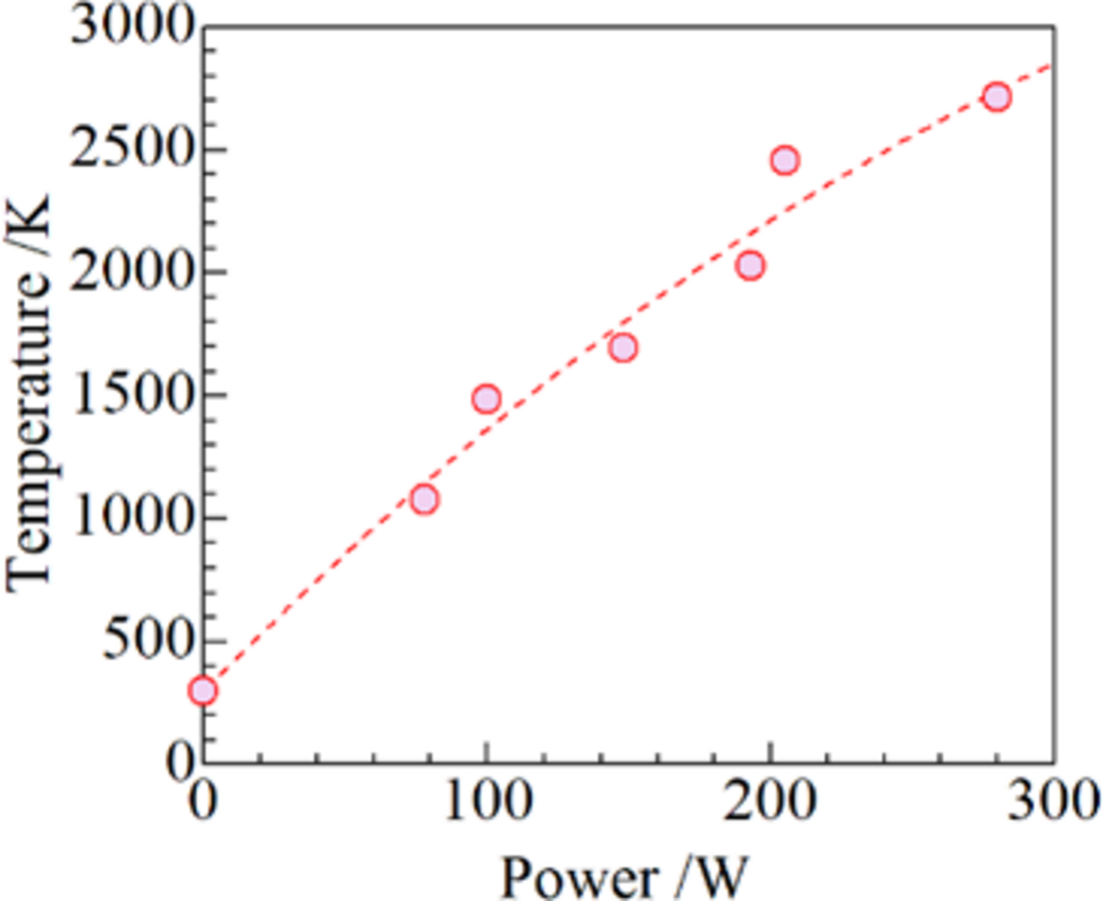
Temperatures estimated using the fitting curve from Swamy *et al.* (1999 ▸) versus power with a fitting curve (dashed line).

Unit-cell parameters up to *P* = 205 W were used to estimate the temperatures applied in this study. Fig. 3 ▸ shows the unit-cell parameters of cubic Y_2_O_3_ at 300–2530 K as a function of temperature, as investigated by Swamy *et al.* (1999 ▸). The temperatures at each power level were estimated, and the obtained values are listed in Table 1 ▸. By adding the melting point of Y_2_O_3_ at 282 W, the following second-order polynomial was obtained,
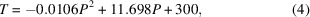
where *T* is the temperature and *P* is the power applied to the specimen. The estimated temperatures against the power using the fitting curve are shown in Fig. 4 ▸.

**Figure 5:**
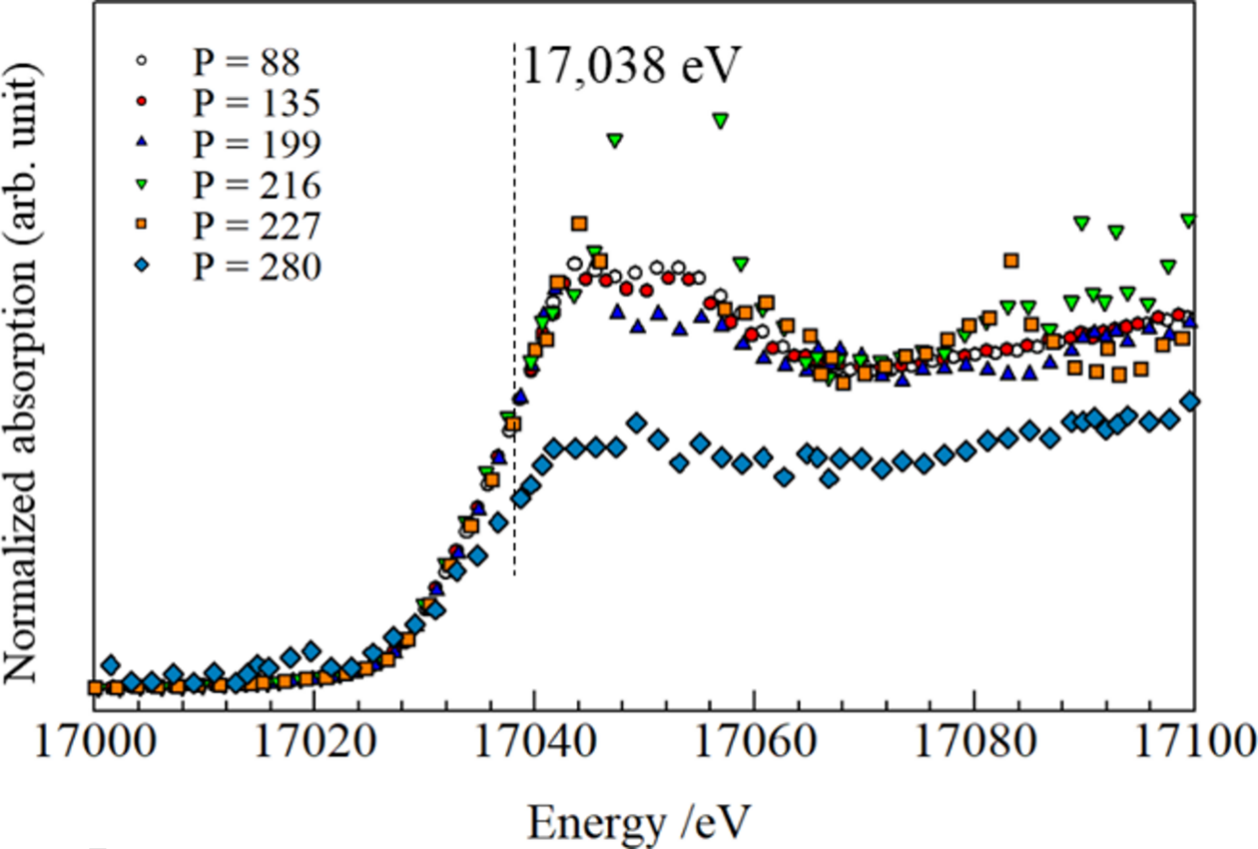
Normalized absorption spectra (Y *K*-edge) at powers of 88, 135, 199, 216, 227 and 280 W.

**Figure 6:**
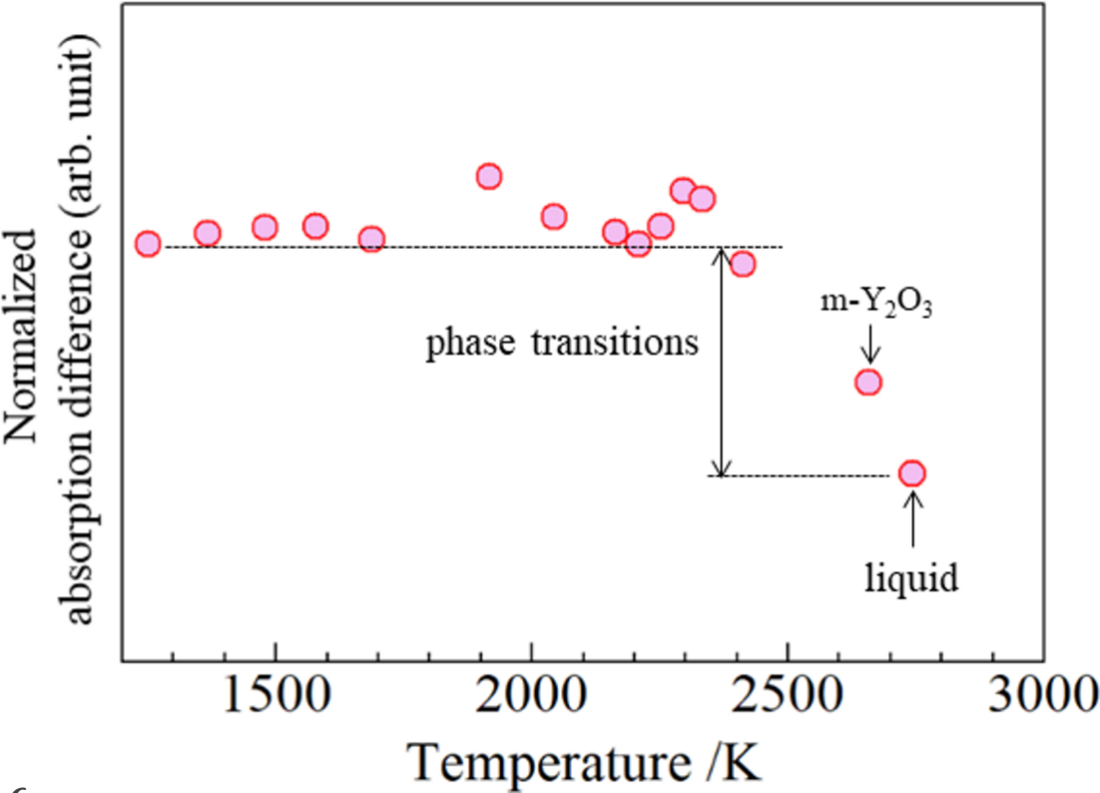
Normalized absorption difference at 17038 eV against calculated temperatures.

The phase transition can be distinguished by examining the absorption difference in the XANES spectra using the T-scan method (Boccato *et al.*, 2020 ▸) in which the absorption coefficient discontinuously dropped due to a drastic change of absorbing atom density. Fig. 5 ▸ shows selected X-ray absorption spectra, indicating that the Yh structure also exhibited low absorption (*P* = 280 W). The absorption differences from 300 K at 17.038 keV are plotted against the temperature in Fig. 6 ▸. The absorption level was almost constant up to 2400 K and then discontinuously decreased at the points where monoclinic Y_2_O_3_ appeared and melted, which is consistent with the observed diffraction pattern.

### 3.2. XAFS–XRD measurement for Zr–Y_2_O_3_

#### 3.2.1. XRD profiles and SEM/TEM of the cooled sample

**Figure 7:**
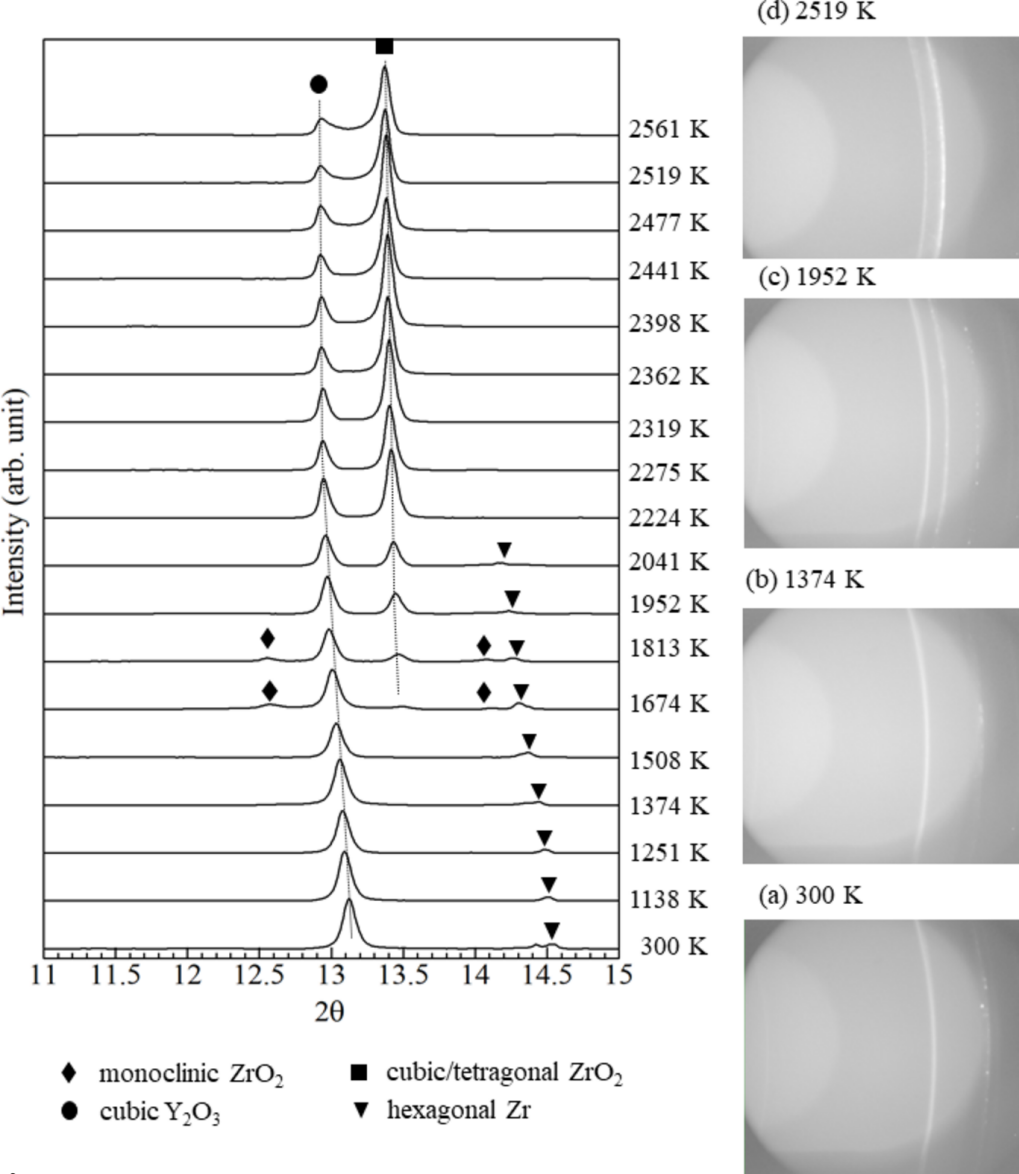
XRD spectra for Zr–Y_2_O_3_ during the heating process, and diffraction images at (*a*) 300 K, (*b*) 1374 K, (*c*) 1952 K and (*d*) 2519 K.

**Figure 8:**
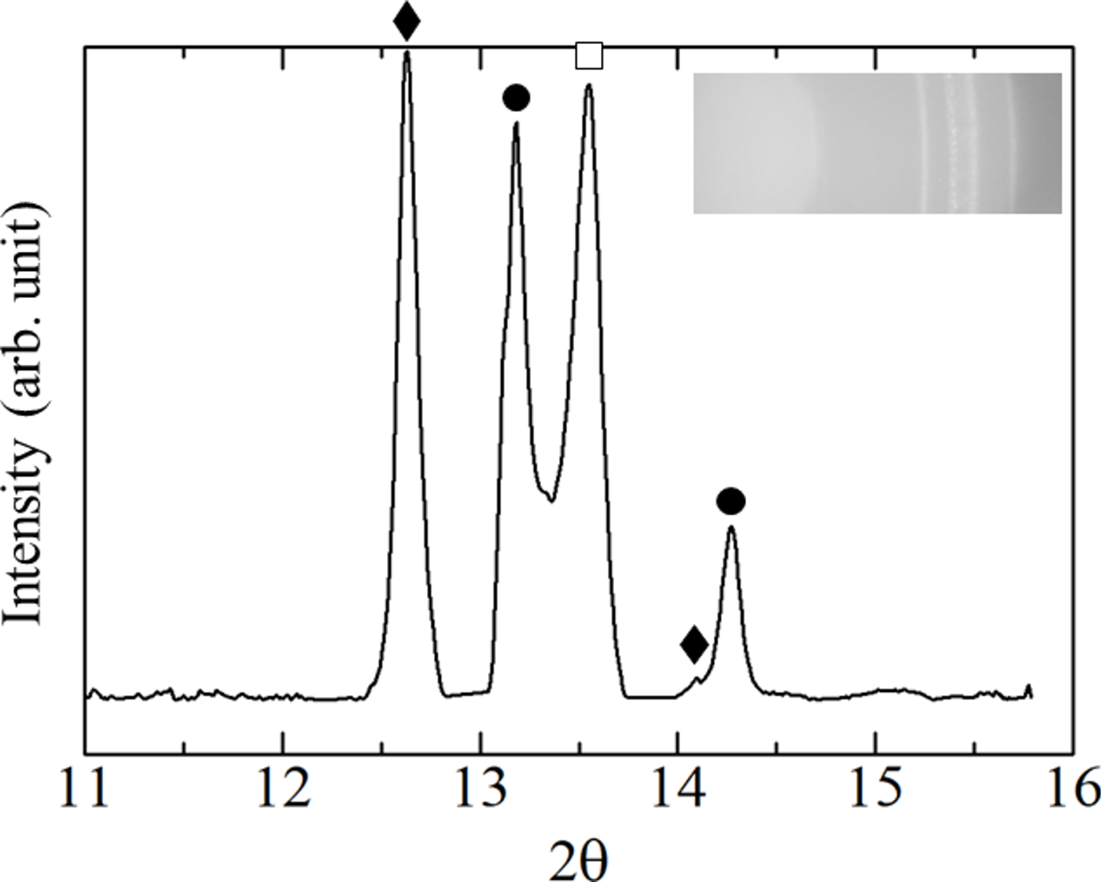
XRD pattern of the cooled sample (black diamonds: monoclinic ZrO_2_; black circles: cubic Y_2_O_3_; white squares: cubic ZrO_2_).

**Figure 9:**
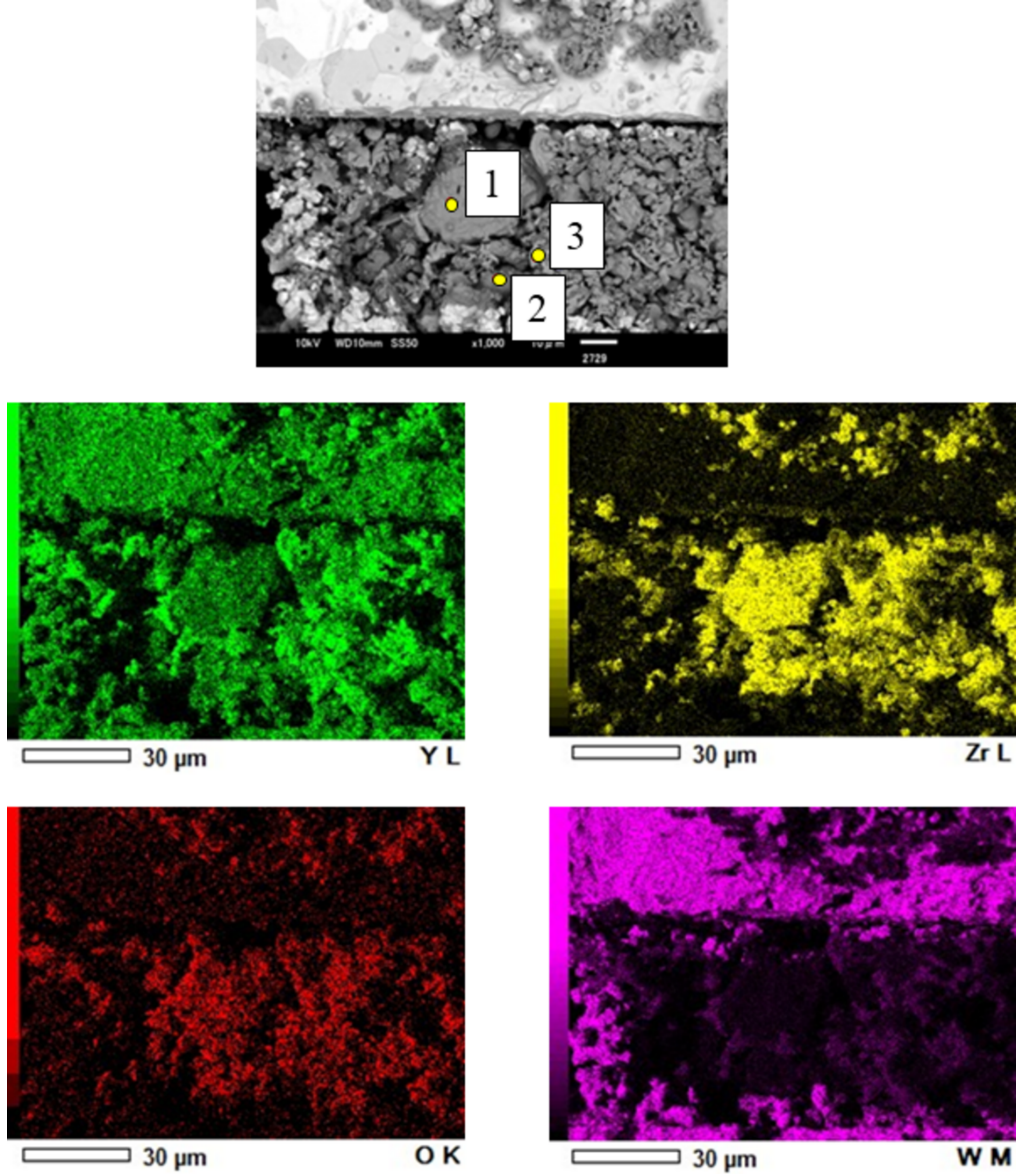
SEM/EDS mapping of the cooled specimen (1: Y_2_O_3_–ZrO_2_ solid solution; 2: ZrO_2_; 3: Y_2_O_3_) at Y-*L* (green), Zr-*L* (yellow), O-*K* (red) and W-*M* (pink).

Fig. 7 ▸ shows XRD profiles obtained by conversion of diffraction images from 300 to 2561 K. The temperatures were converted to Kelvin using equation (4). Before heating at 300 K, peaks corresponding to cubic Y_2_O_3_ and hexagonal Zr were observed. In the diffraction image, a continuous ring was clearly observed for cubic Y_2_O_3_, whereas a spotted ring was observed for hexagonal Zr [Fig. 7 ▸(*a*)]. As the temperature increased, the Zr peak became less intense [Fig. 7 ▸(*b*)] and disappeared at 2224 K, whereas the peak of tetragonal ZrO_2_ appeared at 1674 K and increased with a decrease in the amplitude of the cubic Y_2_O_3_ peak. Fig. 7 ▸(*c*) shows the moment at 1952 K at which cubic ZrO_2_ peak became stronger whereas cubic Y_2_O_3_ and hexagonal Zr peaks became weaker. The diffraction image at 2519 K [Fig. 7 ▸(*d*)] shows spots between the two rings (cubic Y_2_O_3_ and ZrO_2_), giving a broad peak in the XRD profile and indicating partial formation of a solid solution of Y_2_O_3_–ZrO_2_ (Butz *et al.*, 2006 ▸). Fig. 8 ▸ shows the XRD pattern obtained after heating test by switching off the current heating, consisting of patterns from cubic Y_2_O_3_, cubic ZrO_2_ and monoclinic ZrO_2_. SEM/EDS analysis of the cooled sample was performed, as shown in Fig. 9 ▸. Three distinct regions were observed: Y–Zr oxide (point 1), Zr-rich oxide (point 2) and Y-rich oxide (point 3). The composition at each point was analyzed by EDS as summarized in Table 2 ▸.

**Figure 10:**
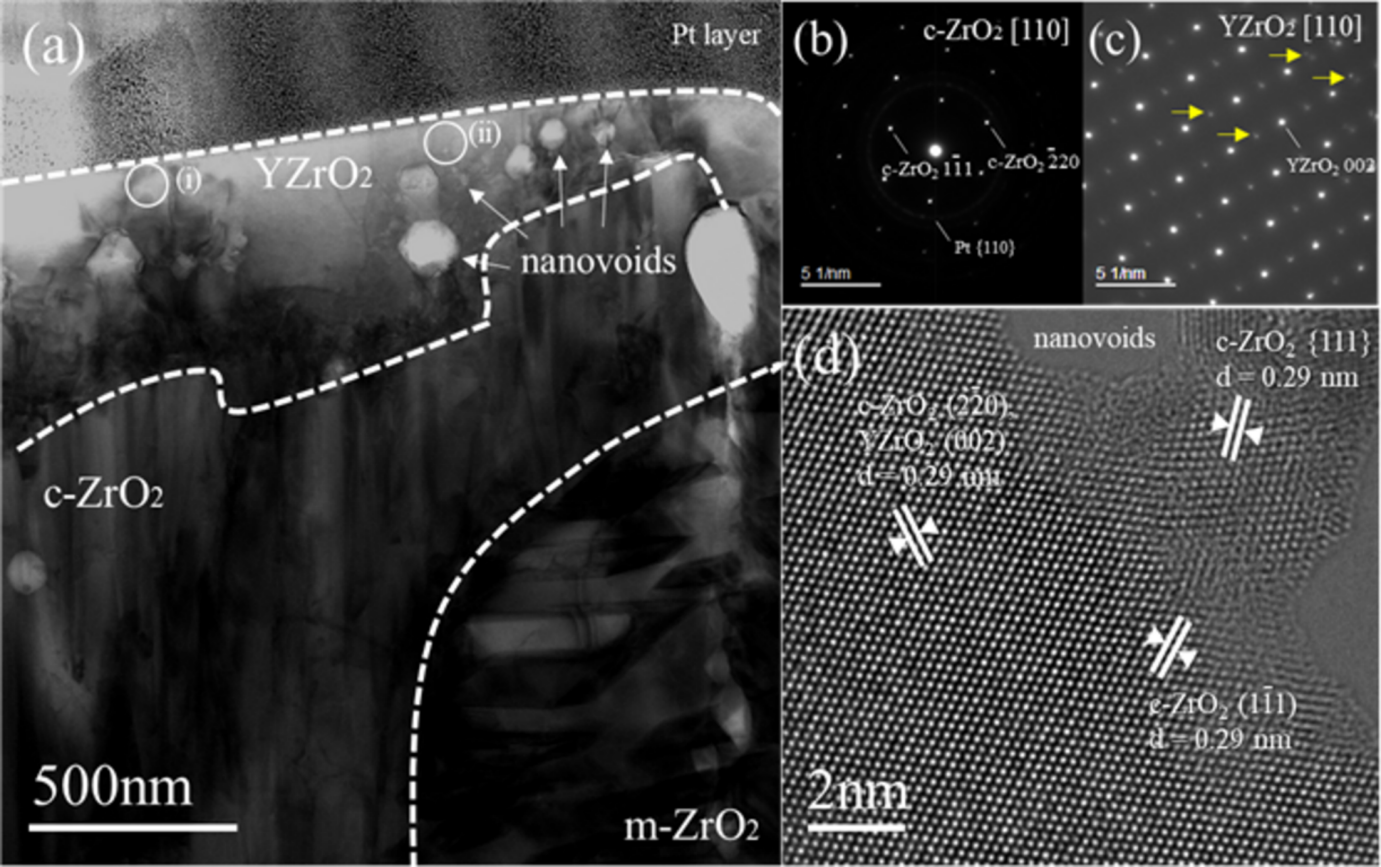
TEM of the cooled specimen (1: Y_2_O_3_–ZrO_2_ solid solution). (*a*) Low-magnification TEM image, (*b* and *c*) selected area diffraction patterns at (i) and (ii), respectively, (*d*) AC-TEM with Cs (spherical aberration coefficient) = 0 nm and Δ*f* = 5 nm.

The TEM analysis of the cooled sample is summarized in Fig. 10 ▸. A membrane sample of thickness 150 nm was fabricated using a focused ion milling technique from a large crystal grain corresponding to the point 1: Y_2_O_3_–ZrO_2_ solid solution, which is shown in Fig. 9 ▸. Figs. 10 ▸(*b*) and 10 ▸(*c*) are selected areas where diffraction patterns were obtained at regions (i) and (ii) in a bright-field TEM image shown as Fig. 10 ▸(*a*). It was found that the annealed coarse grain was mainly composed of monoclinic ZrO_2_ polycrystalline grains and cubic ZrO_2_ polycrystalline grains. In addition, near the surface of the annealed coarse grain, cubic YZrO_2_ aggregation containing many nanovoids was observed. Fig. 10 ▸(*d*) is an aberration-corrected TEM (AC-TEM) image along the [110] direction of the cubic YZrO_2_, which shows an extra spot in the diffraction pattern in Fig. 10 ▸(*c*) (indicated by the arrow). Lattice distances such as *d* = 0.29 nm and 0.18 nm were almost equal to ones in other cubic ZrO_2_ grains.

#### 3.2.2. Chemical shift of the Zr-*K* absorption spectra

**Figure 11:**
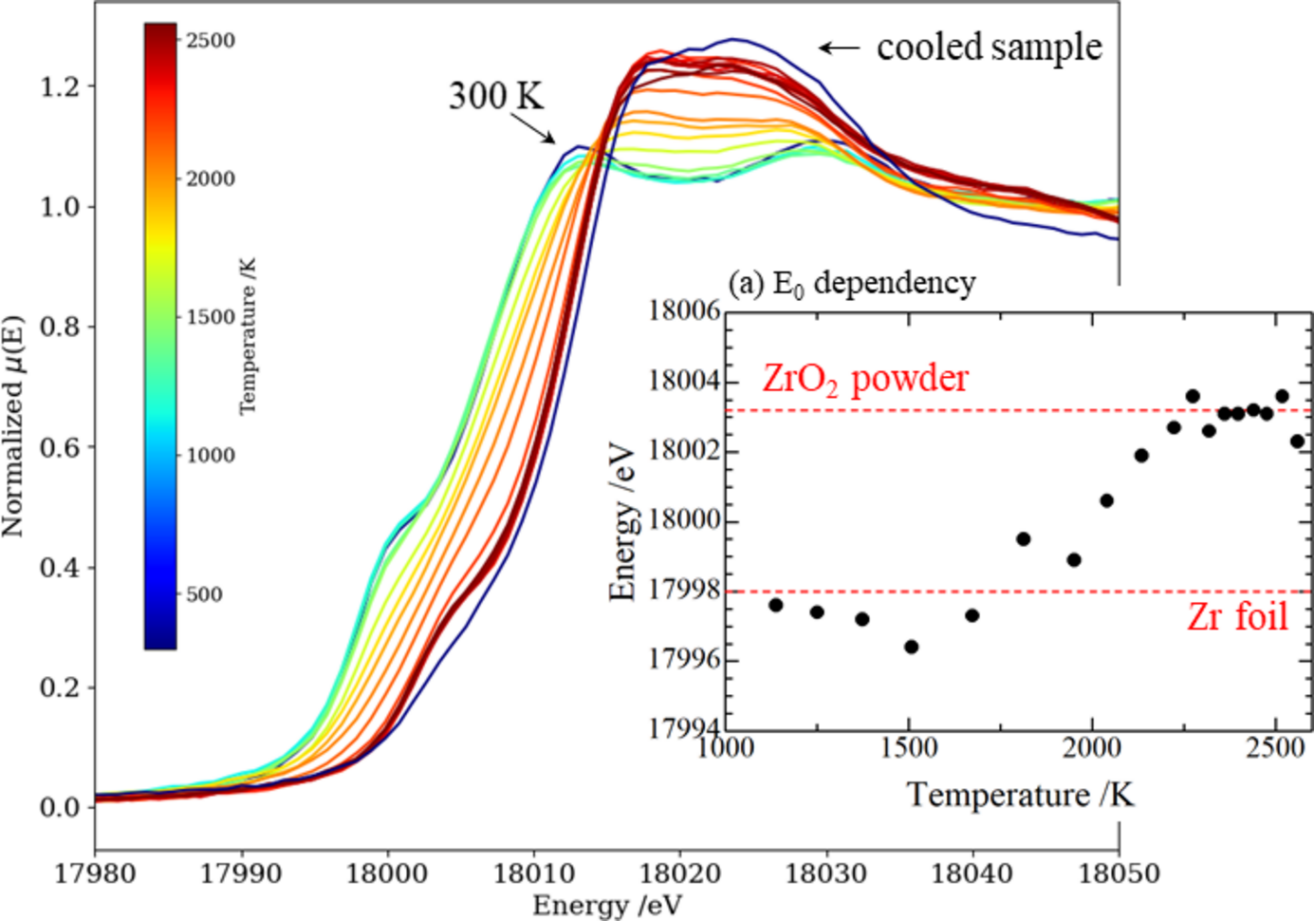
Normalized absorption spectra at the Zr *K*-edge and (*a*) the dependency of *E*_0_ on temperature.

The normalized X-ray absorption coefficient was plotted against the energy of the incident X-rays for each temperature as shown in Fig. 11 ▸. The value of *E*_0_ at 300 K was calibrated by the XAFS spectrum of Zr foil (99.99% purity, Nilaco Co. Ltd) at 17.998 keV. The temperature dependency of *E*_0_ is plotted in Fig. 11 ▸(*a*), showing that *E*_0_ remained at the level of Zr until 1674 K, shifted continuously to the higher energy range with increasing of temperature to 2041 K, reached the same level as the ZrO_2_ powder above 2200 K, and then settled at this level thereafter.

#### 3.2.3. EXAFS spectra during heating and after cooling

**Figure 12:**
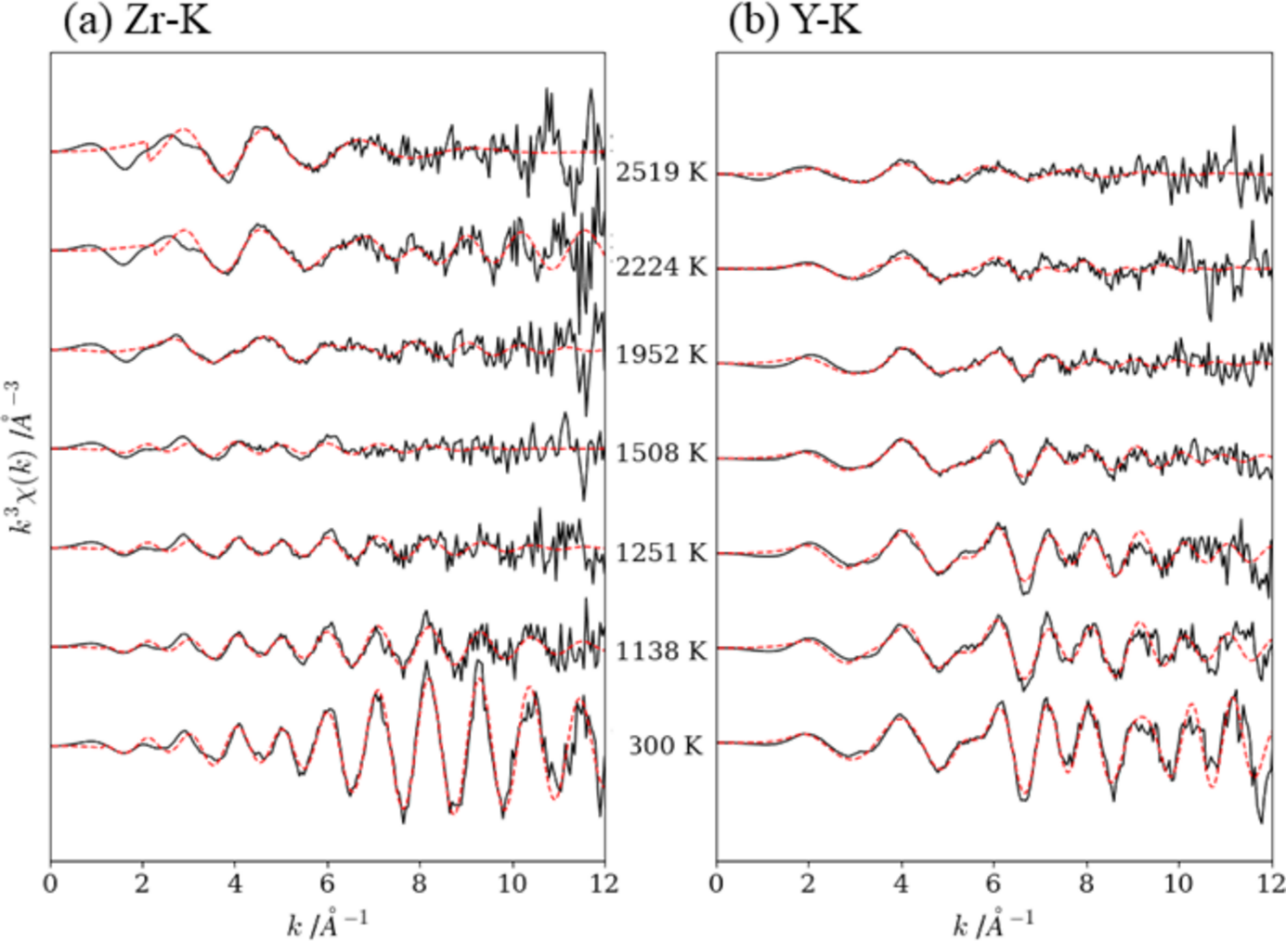
Fits between experimental (solid lines) and calculated (dots) *k*^3^-weighted χ(*k*) at (*a*) Zr-*K* and (*b*) Y-*K*.

**Figure 13:**
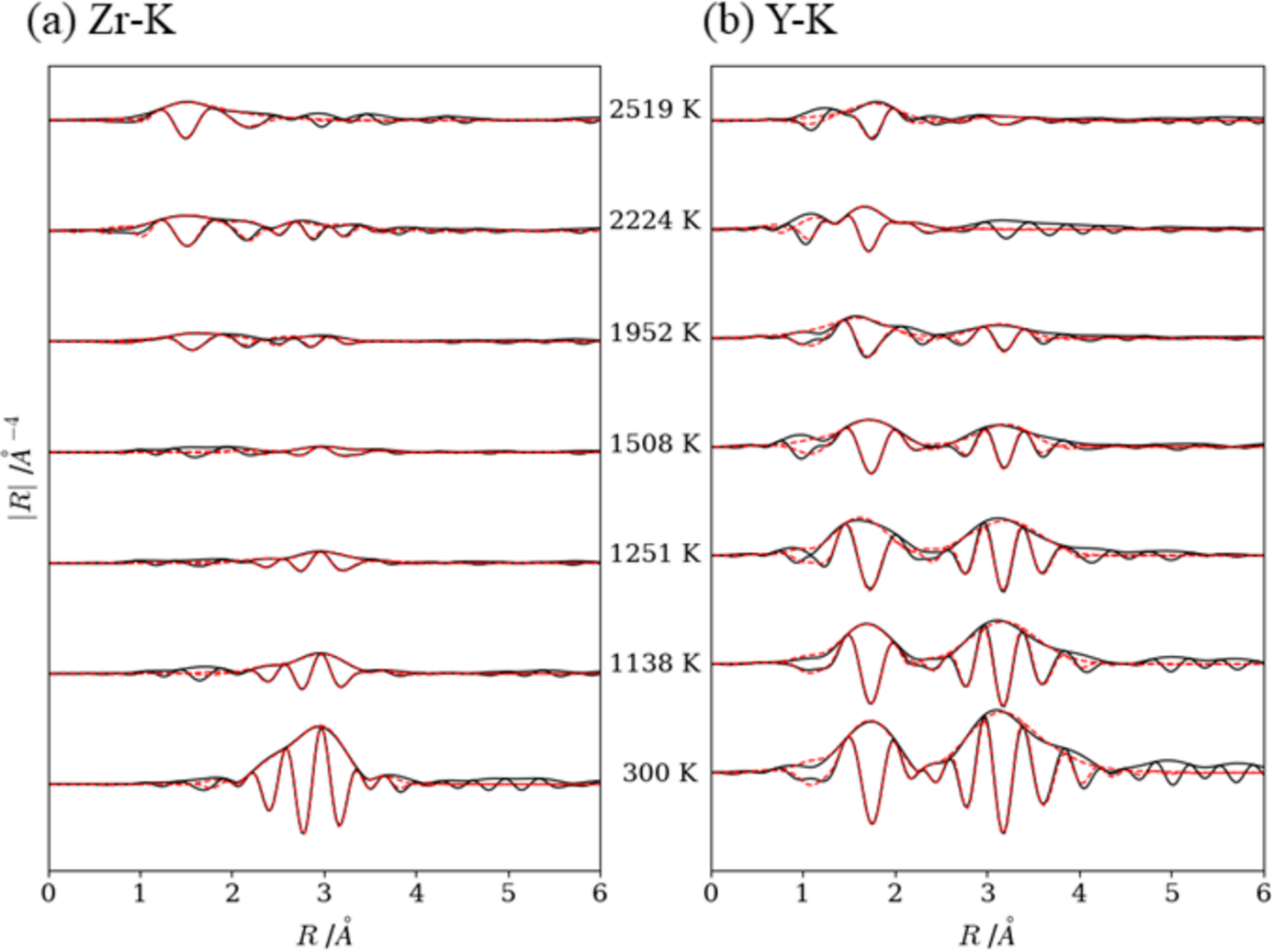
Fits during the heating process between experimental (solid lines) and calculated (dots) Fourier transformed with the imaginary part at (*a*) Zr-*K* and (*b*) Y-*K*.

We investigated the EXAFS spectra to understand the local structural changes during the heating process. Extracted EXAFS oscillations for Zr and Y at the *K*-edge with fitting results at 300, 1138, 1251, 1508, 1952, 2224 and 2519 K are plotted in *k*-space as shown in Fig. 12 ▸. With the exception of 300 K, the *k*^3^-weighted oscillations were too large to provide appropriate fitting results in the range greater than *k* = 10. Hence, the range of the Fourier transform was set to the *k*-range of 3–10 to calculate the RSF (Fig. 13 ▸). The curve fitting analysis results are summarized in Table 3 ▸.

The RSF for Zr-*K* at 300 K exhibited the first peak at ∼2.9 Å, which is attributable to the contribution of the first coordination shell (Zr—Zr) with the bonding distance of 3.21 Å. The RSF for Y-*K* exhibited the first peak at ∼1.7 Å owing to the first coordination shell (Y—O, nearest six oxygen atoms) and the second peak at ∼3.58 Å mainly owing to the second coordination shells (Y—Y). Cubic Y_2_O_3_ exhibits a variety of peaks owing to the non-equivalent Y atoms, Y1 and Y2 (Jonane *et al.*, 2016 ▸). Out of two types of Y—O coordination, one exhibits six equivalent bonding lengths of 2.89 Å and the other exhibits distorted octahedral structure with three groups of slightly different bonding lengths of (2.24, 2.27, 2.33 Å) × 2. The averaged value of the latter groups is 2.27 Å, which was 1.7% smaller than the value of the present study (2.31 Å).

As the temperature increases, the peak intensity of RSF for Zr—Zr decreases and almost disappears at 1952 K, whereas the appearance of the peak at ∼1.5 Å suggests that the first coordination was changed from Zr—Zr to Zr—O. Regarding the RSF for the Y-*K* edge, the peak intensity of Y—Y decreases above 1508 K and almost disappears at 2224 K.

**Figure 14:**
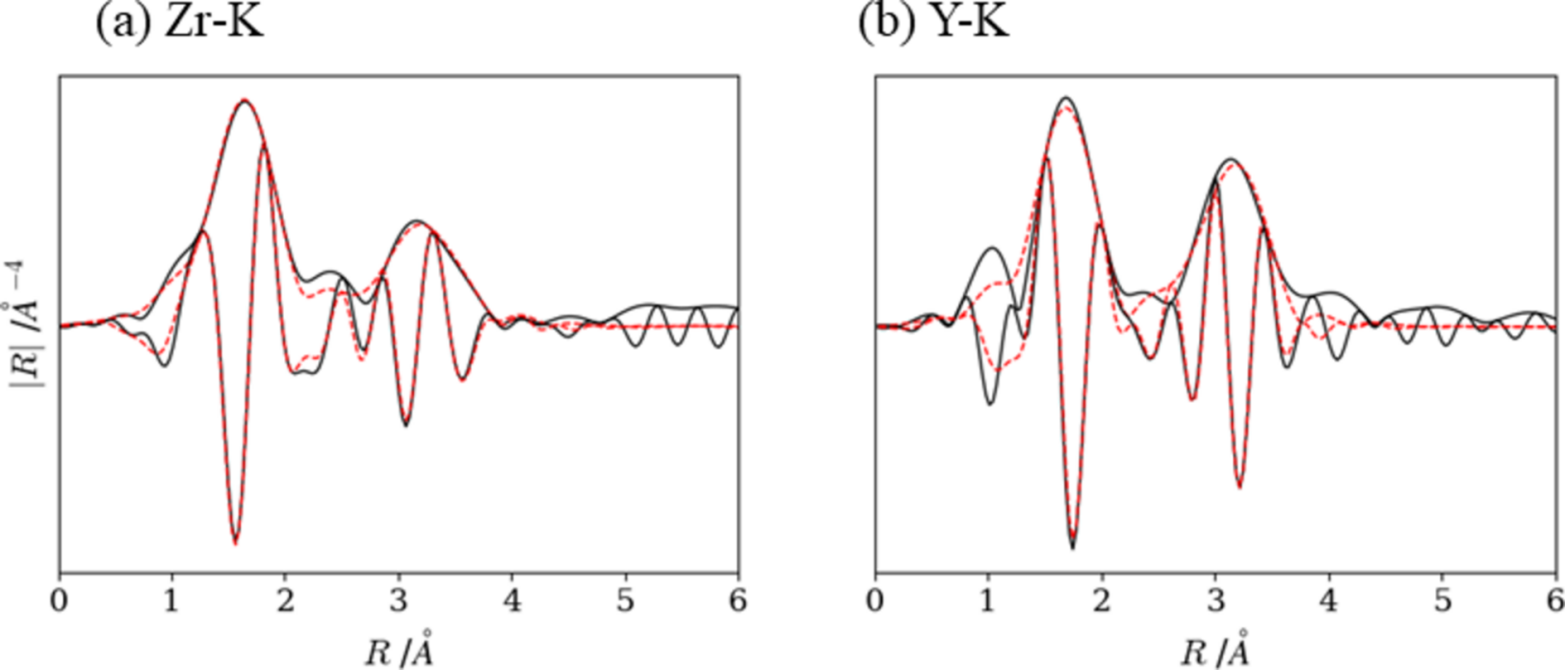
Experimental (solid lines) and calculated (dots) fits of the cooled sample Fourier transformed with the imaginary part. (*a*) Zr-*K* and (*b*) Y-*K*.

Fig. 14 ▸ shows the RSF of the cooled sample after switching off current-heating. The RSF for Zr-*K* exhibits three peaks at ∼1.5, ∼2.5 and ∼3.1 Å, arising from the first coordination shell (Zr—O), the second and third coordination shells (Zr—Zr), respectively. The interatomic distances for each shell are close to the averaged bonding length of Zr—O in monoclinic ZrO_2_ (2.16 Å), Zr—Zr in monoclinic ZrO_2_ (3.44 Å) and Zr—Zr in cubic ZrO_2_ (3.63 Å). The RSF for Y-*K* exhibited several peaks in the *R*-range of 1–6 Å; however, we performed fitting analysis against the second and third peaks from 1.2 to 4 Å because the first peak at ∼1 Å probably appeared as a truncation error of the Fourier transformed calculation. Obtained interatomic distances were 2.33 Å for Y—O and 3.64 Å for Y—Y, respectively, which were slightly larger than the averaged bonding length of cubic Y_2_O_3_.

## 4. Discussion

### 4.1. Phase transitions and local structure change during the heating process

**Figure 15:**
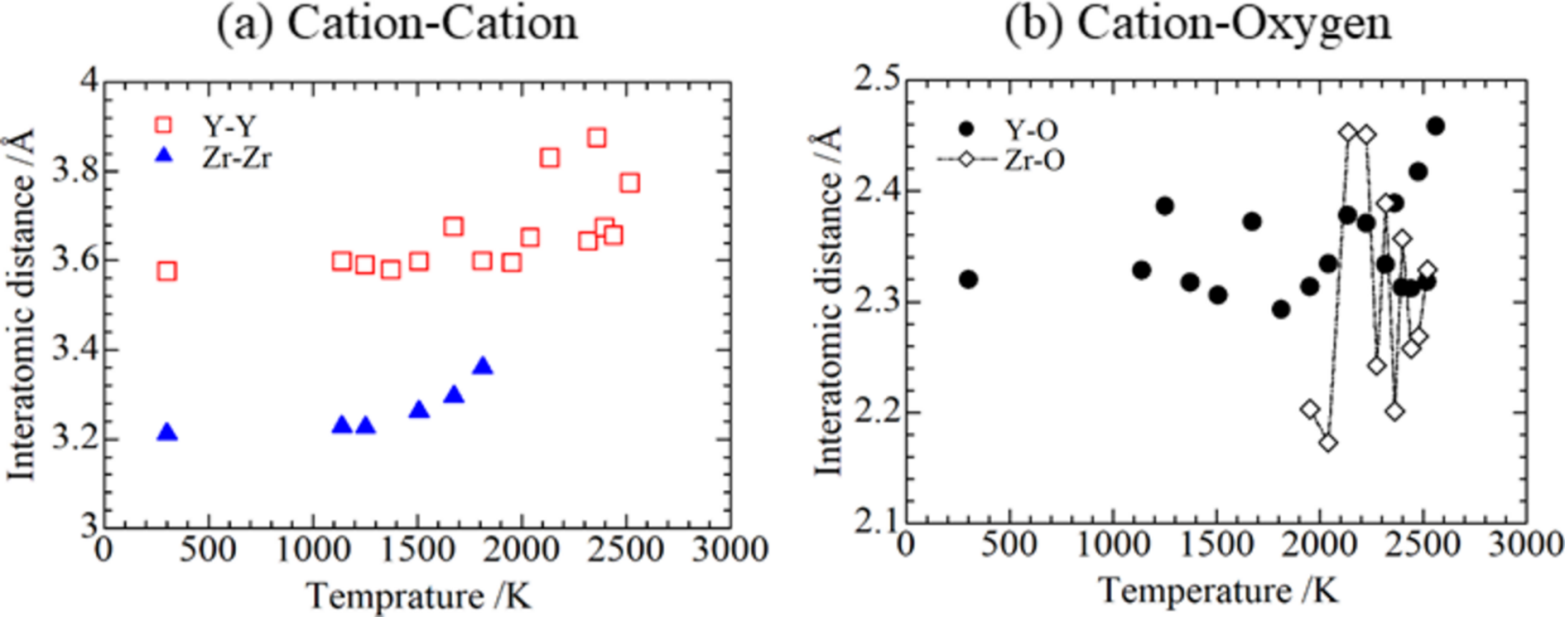
Interatomic distances of the (*a*) cation–cation shell and (*b*) cation–oxygen shell.

Based on the XRD patterns (Fig. 7 ▸), ZrO_2_ was formed above 1674 K and coexisted with Zr up to 2041 K. In this temperature range, the absorption edge *E*_0_ shows a continuous shift to higher energy range, indicating that Zr was gradually oxidized. Since hexagonal Zr can dissolve oxygen up to ∼30 at% according to the Zr—O phase diagram (Arroyave *et al.*, 2002 ▸), oxygen-dissolved Zr [*i.e.* α-Zr(O)] coexisted with ZrO_2_. Hence, the X-ray absorption could have contributions from α-Zr(O) and ZrO_2_, then exhibit a gradual change. This interpretation can explain the interatomic distance of Zr—Zr shown in Fig. 15 ▸(*a*), which gradually increases as temperature increases above 1500 K. Oxidation may have been caused by the residual oxygen in the furnace, not by reducing Y_2_O_3_ because the equilibrium oxygen partial pressure of Y is much lower than that of Zr (Kleykamp, 1985 ▸).

At 1952 K, the reaction between ZrO_2_ and Y_2_O_3_ could have commenced, affecting the RSF of Y-*K* in which the peak for Y—Y shells mostly disappeared. Tuilier *et al.* (1987 ▸) observed similar behavior when investigating the Y_2_O_3_–ZrO_2_ reaction annealed at 1700–1800°C. Above 1952 K, the interatomic distance of Y—Y and Y—O obtained by the EXAFS analysis shows increasing tendency with large fluctuation [Figs. 15 ▸(*a*) and 15(*b*)] and the coordination number of Y—Y decreased from 12.0 to 8.6 (Table 3 ▸). The Zr—O distance [Fig. 15 ▸(*b*)] also shows large fluctuations, which may arise from the low S/N ratio of the measurement data. The constraint for fitting of the third-order cumulant could have affected the interatomic distance, causing such fluctuation. This result was also observed in the study of ZrO_2_-doped yttria (Duraud *et al.*, 1990 ▸; Thromat *et al.*, 1991 ▸). Those authors suggested that a small value of the second cumulant and a large oxygen deficit around Y contradicted the theory of vacancy formation in Y_2_O_3_-doping into ZrO_2_ [it is well known that when Y_2_O_3_ is dissolved in ZrO_2_ an oxide defect could be formed to compensate for the electrical charge because the trivalent Y has a negative charge on the tetravalent Zr, and the oxide defects tend to gather around Zr, not Y (Ando & Oishi, 1985 ▸)]. Others investigated the site selectivity in ZrO_2_-doped Y_2_O_3_ by spectra calculation (Crocombette & Jollet, 1994 ▸), stating that Zr could tend to more probably substitute for Y2 than for Y1, although the relation between their conclusion and the large oxide defect for Y—O is still unknown. This different observation between ZrO_2_-doped Y_2_O_3_ and Y_2_O_3_-doped ZrO_2_ could be caused by a different defect formation mechanism. The possibility of an oxygen deficit around the metal atom in ZrO_2_-doping reactions is important in our target system (U—Zr—O) because the interaction between metal and oxygen-deficient metal could promote eutectic melting. From this point of view, the present study is valuable for future work.

### 4.2. Influence of anharmonic phonon oscillations on the local structure

At high temperature, anharmonic effects in the interatomic potential cannot be neglected in temperature-dependent EXAFS studies. In the present study, the temperature dependencies of the obtained second- and third-order cumulants were analyzed by comparison with the theoretical ones proposed by Miyanaga & Fujikawa (1994 ▸), who calculated the temperature dependency of the second- and third-order cumulant for the diatomic model in which the Morse potential, *V*(*x*), is assumed as the interatomic potential, as
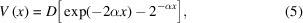

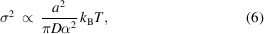

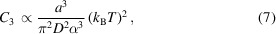
where σ^2^ is the second-order cumulant, *C*_3_ is the third-order cumulant, *D* and α are parameters in the Morse potential, *a* is the interatomic distance between the absorbing and scattering atoms, *k*_B_ is the Boltzmann constant and *T* is temperature. As described in the equations, σ^2^ arising mainly from harmonic phonon oscillation varies linearly on *T* reflecting attenuation of the EXAFS amplitude, while *C*_3_ from anharmonic effects depends on *T*^2^ reflecting the asymmetry of the interatomic distance distribution (deviation from the symmetric Gaussian distribution).

**Figure 16:**
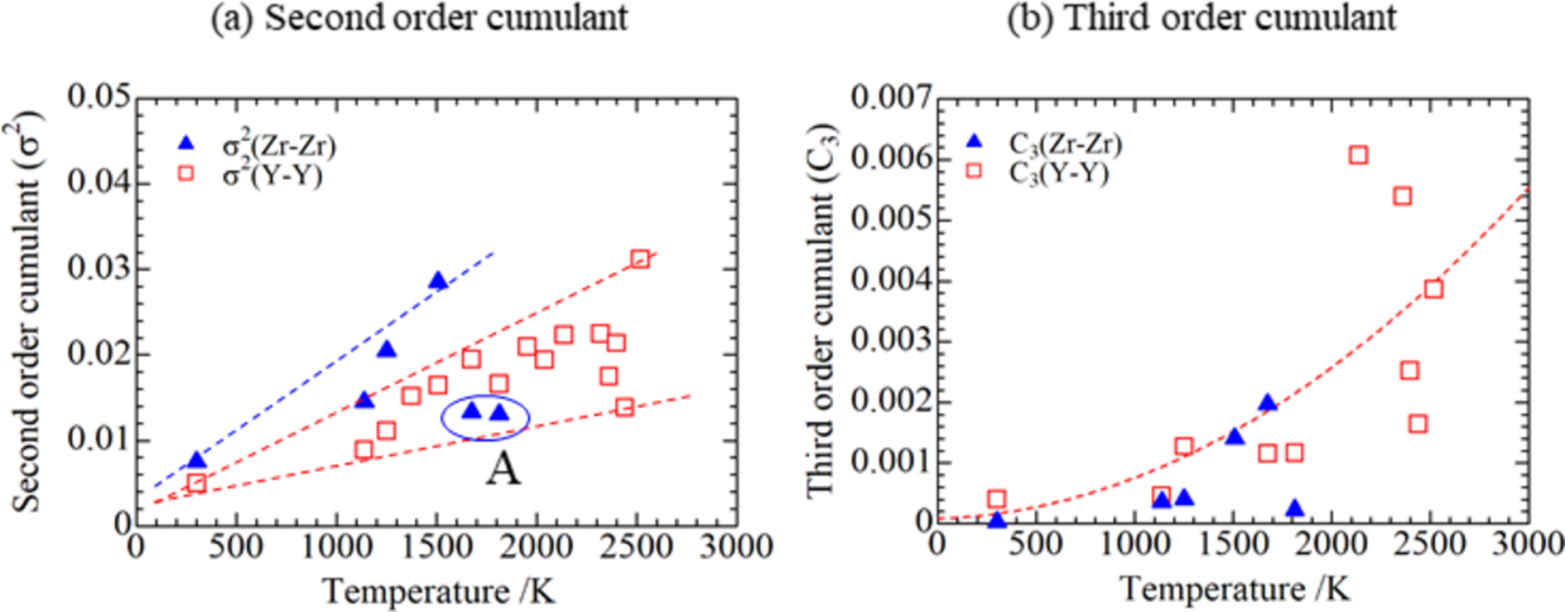
Temperature dependence of the (*a*) second-order cumulant and (*b*) third-order cumulant for the cation–cation shell.

**Figure 17:**
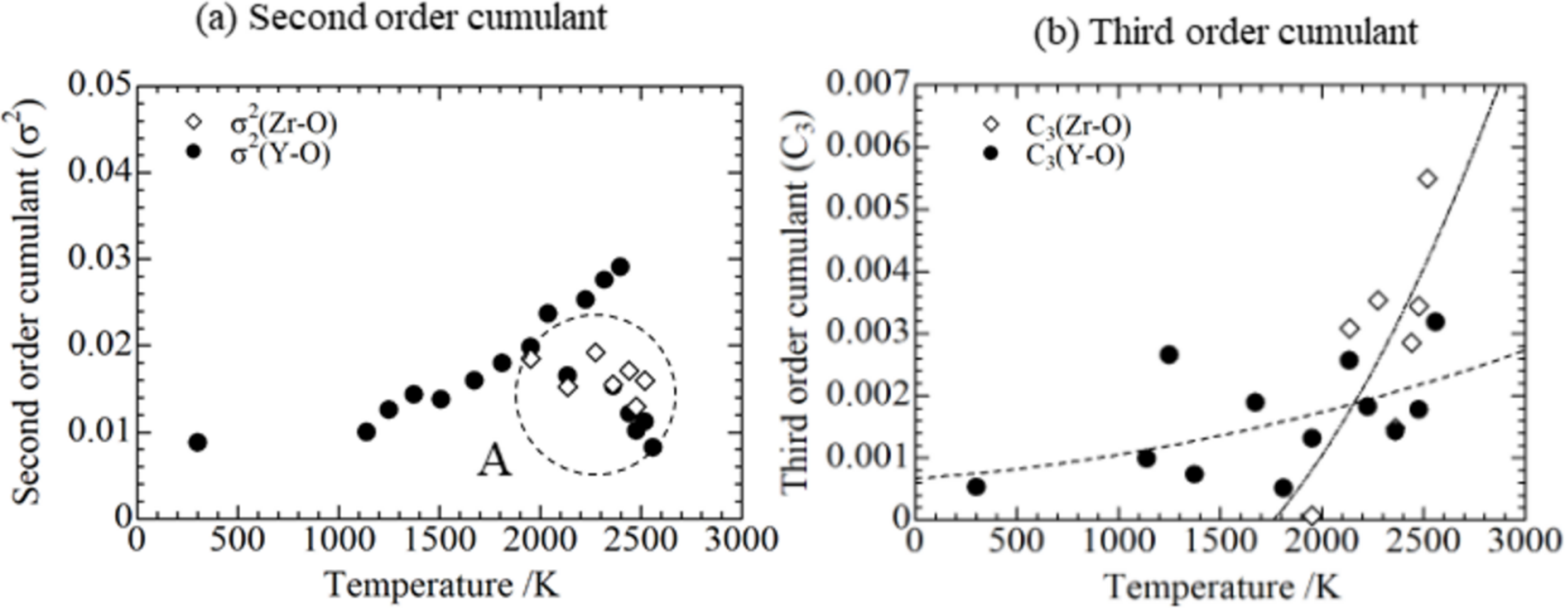
Temperature dependence of the (*a*) second-order cumulant and (*b*) third-order cumulant for the cation–oxygen shell.

Figs. 16 ▸ and 17 ▸ show the variation of σ^2^ and *C*_3_ with *T* for cation–cation and cation–oxygen couples. For the cation–cation couples, σ^2^ of Y—Y tended to increase linearly, whereas that of Zr—Zr increased linearly up to approximately 1500 K and then decreased [Fig. 16 ▸(*a*) circled as A]. This may be because oxidation of Zr somehow stabilized Zr—Zr bonding. The values of *C*_3_ exhibited the tendency to increase as shown in Fig. 16 ▸(*b*). Positive values of the third-order cumulant means that the interatomic distance distribution has more weight on the longer bonds. Hence, this behavior is consistent for longer interatomic distances at high temperatures. For the cation–oxygen couples, σ^2^ for Y—O clearly showed a tendency to linearly increase up to approximately 2000 K, whereas σ^2^ of Zr—O and Y—O above 2000 K tended to decrease [Fig. 17 ▸(*a*) circled as A], although some points for Y—O showed a tendency to increase. This behavior might be attributable to stabilization of bonds due to the ZrO_2_–Y_2_O_3_ reaction. On the other hand, as shown in the cation–cation shell, *C*_3_ of Y—O and Zr—O showed an increase with temperature. This is also consistent with the tendency shown in the interatomic distance for the cation–oxygen shell which increase as temperature increased. Based on these observations, the fits applying the diatomic theory proposed by Miyanaga & Fujikawa (1994 ▸) qualitatively explained the high-temperature interaction in the present study. Also, it was found that thermal disorder above 2000 K from the ZrO_2_–Y_2_O_3_ reaction was caused mostly by anharmonic effects.

### 4.3. State of the cooled sample after the heating process

The specimen was rapidly quenched by switching off the current-heating. Therefore, part of the high-temperature state could have remained in the cooled sample. The SEM image in Fig. 9 ▸ shows three distinct regions: Y–Zr oxide (point 1), Zr-rich oxide (point 2) and Y-rich oxide (point 3), which is consistent with the XRD patterns of the cooled sample as shown in Fig. 8 ▸. The broad peak-top splitting pattern from 13.17 to 13.54° suggests that the Y_2_O_3_–ZrO_2_ reaction formed polycrystalline forms of (Y, Zr)O_2–*x*_ solid solution with various Y/Zr ratios, which is also consistent with the TEM analysis result as shown in Fig. 10 ▸(*a*). A number of lattice distortion and defects were observed in the cubic YZrO_2_ grain, especially near nanovoids. Since an extra spot in Fig. 10 ▸(*c*) (noted by the arrow) was a point which did not appear by the extinction rule, the YZrO_2_ structure was probably not a ‘perfect cubic’ structure (Butz *et al.*, 2006 ▸). This result means that some of the Y_2_O_3_ powder was dissolved and mixed with ZrO_2_ during heating, and that many vacancies around the yttrium solid solution atoms existed after cooling. A large oxygen deficit around the Y atom and eight O atoms coordinated around Zr may be caused by this metallographic structure.

### 4.4. Applicability of the developed system to the UO_2_–Zr reaction

In the present study, we succeeded in simultaneous XAFS–XRD measurements of reaction processes in metals and metal-oxides at ultra-high temperatures above 2000 K. The structural changes in which Zr was oxidized by the impurity oxygen in Ar gas and the ZrO_2_–Y_2_O_3_ reaction proceeded were successfully captured. Since the specimen was very small, it was difficult to measure the temperature using a pyrometer. To solve this problem, temperature calibration was performed using the diffraction pattern of Y_2_O_3_, for which there is some knowledge on the temperature dependence of the lattice parameter at high temperatures. Although this method has the limitation that the same equation cannot be used if the heat transfer characteristics of the sample holder change, we think that this method could be applied to different combinations of materials if temperature calibration is performed against the oxide material in advance. Also, in the present study, Zr was oxidized by residual oxygen in the gas phase. To analyze the melting process of UO_2_–Zr, the oxygen partial pressure in the furnace must be controlled at a sufficiently low level.

The quality of the obtained spectra was acceptable for EXAFS analysis, although the signal-to-noise ratio in the high-energy region tends to deteriorate above ∼2000 K. For diffraction images, it is desirable to be able to scan transmitted X-rays at higher angles to improve the distinguishability of cubic or tetragonal oxide. This can be overcome by improving the equipment, such as widening the glass window at the exit of the chamber’s X-ray transmission window. Thus, by implementing some device modifications, it is possible to apply this measurement technique to UO_2_–Zr mixtures and investigate the structural changes leading to the melting process.

## 5. Conclusion

*In situ* simultaneous XAFS–XRD measurements of Y_2_O_3_ and Zr–Y_2_O_3_ at high temperatures above 2000 K were performed. The XAFS signal was taken in transmission mode for the Zr *K*- and Y *K*-edges, and the transmitted X-rays were captured by CCD camera as diffraction images. By combining analysis of the XRD patterns and EXAFS spectra, we succeeded in capturing the structure changes during the Zr oxidation up to 1952 K and the interaction between ZrO_2_ and Y_2_O_3_ for temperatures from 1952 and 2519 K. The interatomic distances obtained by the EXAFS fitting analysis were confirmed to agree with the literature data for the ZrO_2_–Y_2_O_3_ system. Also, we investigated the temperature dependence of the anharmonic effect by comparing the fitted second- and third-order cumulants, leading to the conclusion that thermal disorder was mainly caused by anharmonic effects during the Y_2_O_3_–ZrO_2_ reaction. Consequently, the developed system in the present study was confirmed to be applicable for investigation of the metal/metal-oxide mixture at extra-high temperature. For application to the melting of nuclear fuel material, although some modification on the equipment will be required, it is possible to adapt this technique for investigation of the UO_2_–Zr reaction leading to the melting process as future work.

## Supplementary Material

Figures S1 to S3. DOI: 10.1107/S1600577524003321/yn5107sup1.pdf

## Figures and Tables

**Table 1:** Unit-cell parameter *a* of c-Y_2_O_3_ as a function of power and calculated temperature

*P* (W)	*d* (Å)	*a* (Å)	*T* (K)
0	3.058	10.6031	300
78	3.076	10.6655	1077
100	3.088	10.7070	1483
148	3.095	10.7313	1696
193	3.107	10.7728	2163
205	3.124	10.8317	2455

**Table 2:** EDS point analysis results of the cooled Zr–Y_2_O_3_ specimen in at%

Position	O	Y	Zr	W
1	60.6	6.2	32.7	0.5
2	71.3	0.9	27.7	0.1
3	57.1	34.3	2.1	6.5

**Table 3:** EXAFS fitting analysis results (for selected temperatures)

Edge	Temperature (K)	Cation–Cation				Cation–Oxygen			
		*R* (Å)	*N*	σ^2^ (×10^−3^)	*C*_3_ (×10^−4^)	*R* (Å)	*N*	σ^2^ (×10^−3^)	*C*_3_ (×10^−4^)
Zr	300	3.21 (1)	12.0 (0)	7.5 (0)	0.29 (0)	–	–	–	–
	1138	3.22 (8)	9.0 (5)	14.5 (5)	3.55 (6)	–	–	–	–
	1251	3.22 (6)	9.1 (4)	20.5 (3)	4.07 (7)	–	–	–	–
	1508	3.26 (1)	8.5 (8)	28.5 (7)	14.03 (1)	–	–	–	–
	1952	–	–	–	–	2.20 (2)	3.2 (2)	18.5 (2)	0.72 (2)
	2224	–	–	–	–	2.45 (1)	3.6 (9)	11.9 (4)	88.79 (8)
	2519	–	–	–	–	2.32 (8)	4.9 (1)	15.9 (7)	54.94 (2)
	Cooled	3.41 (0)	7.5 (3)	0.2 (5)	2.60 (7)	2.16 (1)	8.9 (9)	10.8 (3)	2.25 (2)
		3.65 (4)	11.2 (9)	1.8 (2)	15.55 (7)				

Y	300	3.57 (3)	6.0 (0)	5.0 (0)	3.98 (1)	2.31 (9)	6.0 (0)	8.8 (0)	5.31 (7)
		4.06 (8)	6.0 (0)						
	1138	3.58 (4)	6.7 (5)	8.8 (5)	4.59 (7)	2.32 (8)	5.0 (4)	10.0 (1)	9.90 (5)
		4.06 (8)	4.3 (2)						
	1251	3.64 (0)	7.1 (1)	11.1 (5)	12.76 (4)	2.38 (6)	5.6 (6)	12.5 (8)	26.61 (7)
		4.10 (8)	4.4 (0)						
	1508	3.59 (7)	8.8 (0)	16.4 (6)	–	2.30 (5)	4.5 (9)	13.7 (6)	–
	1952	3.59 (4)	8.6 (3)	20.9 (5)	–	2.31 (3)	5.1 (1)	19.8 (3)	13.15 (2)
	2224	–	–	–	–	2.27 (0)	5.5 (7)	25.3 (4)	18.19 (5)
	2519	–	–	–	–	2.31 (7)	2.2 (4)	11.1 (9)	–
	Cooled	3.64 (5)	12.0 (1)	16.1 (7)	6.14 (3)	2.33 (8)	4.1 (8)	7.2 (3)	11.75 (4)

## References

[bb1] Ando, K. & Oishi, Y. (1985). *Transport in Nonstoichiometric Compounds*, pp. 203–215. Boston: Springer.

[bb2] Andrievskaya, E. R., Lopato, L. M. & Smirnov, V. P. (1996). *J. Am. Ceram. Soc.* **79**, 714–720.

[bb3] Arroyave, R., Kaufman, L. & Eagar, T. W. (2002). *Calphad*, **26**, 95–118.

[bb4] Bessada, C., Zanghi, D., Pauvert, O., Maksoud, L., Gil-Martin, A., Sarou-Kanian, V., Melin, P., Brassamin, S., Nezu, A. & Matsuura, H. (2017). *J. Nucl. Mater.* **494**, 192–199.

[bb5] Bessada, C., Zanghi, D., Salanne, M., Gil-Martin, A., Gibilaro, M., Chamelot, P., Massot, L., Nezu, A. & Matsuura, H. (2020). *J. Mol. Liq.* **307**, 112927.

[bb6] Boccato, S., Torchio, R., Anzellini, S., Boulard, E., Guyot, F., Irifune, T., Harmand, M., Kantor, I., Miozzi, F., Parisiades, P., Rosa, A. D., Antonangeli, D. & Morard, G. (2020). *Sci. Rep.* **10**, 11663. 10.1038/s41598-020-68244-3 PMC7363681 32669572

[bb7] Butz, B., Kruse, P., Störmer, H., Gerthsen, D., Müller, A., Weber, A. & Iverstiffee, E. (2006). *Solid State Ion.* **177**, 3275–3284.

[bb8] Crocombette, J. P. & Jollet, F. (1994). *J. Phys. Condens. Matter*, **6**, 8341–8348.

[bb9] Duraud, J., Jollet, F., Thromat, N., Gautier, M., Maire, P., Le Gressus, C. & Dartyge, E. (1990). *J. Am. Ceram. Soc.* **73**, 2467–2473.

[bb10] Hayward, P. J. & George, I. M. (1994). *J. Nucl. Mater.* **208**, 35–42.

[bb11] Hayward, P. J. & George, I. M. (1996). *J. Nucl. Mater.* **232**, 13–22.

[bb12] Hayward, P. J., Hofmann, P., Stuckert, J., Berdyshev, A. V. & Veshchunov, M. S. (1999). *UO_2_ Dissolution by Molten Zircaloy. New Experimental Results and Modelling*. Report FZKA-6379. Forschungszentrum Karlsruhe GmbH, Karlsruhe, Germany.

[bb13] Hofmann, P. & Politis, C. (1979). *J. Nucl. Mater.* **87**, 375–397.

[bb14] Jacobson, N. S., Liu, Z. K., Kaufman, L. & Zhang, F. (2004). *J. Am. Ceram. Soc.* **87**, 1559–1566.

[bb15] Jonane, I., Lazdins, K., Timoshenko, J., Kuzmin, A., Purans, J., Vladimirov, P., Gräning, T. & Hoffmann, J. (2016). *J. Synchrotron Rad.* **23**, 510–518. 10.1107/S1600577516001181 26917139

[bb16] Kim, K. T. & Olander, D. R. (1988). *J. Nucl. Mater.* **154**, 102–115.

[bb17] Kim, Y.-I. & Izumi, F. (1994). *J. Ceram. Soc. Japan*, **102**, 401–404.

[bb18] Kleykamp, H. (1985). *J. Nucl. Mater.* **131**, 221–246.

[bb19] Miyanaga, T. & Fujikawa, T. (1994). *J. Phys. Soc. Jpn*, **63**, 3683–3690.

[bb20] Niino, K., Arita, Y., Konashi, K., Watanabe, H., Yaita, T., Tanida, H., Kobayashi, T., Morimoto, K., Watanabe, M. & Miura, Y. (2023). *J. Nucl. Sci. Technol.* pp. 1–7.

[bb21] Okamoto, Y., Osugi, T., Akabori, M., Kobayashi, T. & Shiwaku, H. (2017). *J. Mol. Liq.* **232**, 285–289.

[bb22] Ravel, B. & Newville, M. (2005). *Phys. Scr.* **T115**, 1007.

[bb23] Rehr, J. J. & Ankudinov, A. L. (2001). *J. Synchrotron Rad.* **8**, 61–65. 10.1107/s0909049500016423 11512868

[bb24] Seto, Y., Nishio-Hamane, D., Nagai, T. & Sata, N. (2010). *Rev. High. Press. Sci. Technol.* **20**, 269–276.

[bb25] Shirasu, N., Sato, T., Suzuki, A., Nagae, Y. & Kurata, M. (2023). *J. Nucl. Sci. Technol.* **60**, 697–714.

[bb26] Shobu, T., Tozawa, K., Shiwaku, H., Konishi, H., Inami, T., Harami, T. & Mizuki, J. (2007). *AIP Conf. Proc.* **879**, 902–906.

[bb27] Skinner, L. B., Benmore, C. J., Weber, J. K. R., Williamson, M. A., Tamalonis, A., Hebden, A., Wiencek, T., Alderman, L. G., Guthrie, M., Leibowitz, L. & Parise, J. B. (2014). *Science*, **346**, 984–987. 10.1126/science.1259709 25414311

[bb28] Skrikanth, V., Sato, A., Yoshimoto, J., Kim, J. H. & Ikegami, T. (1994). *Cryst. Res. Technol.* **29**, 981–984.

[bb29] Swamy, V., Dubrovinskaya, N. A. & Dubrovinsky, L. S. (1999). *J. Mater. Res.* **14**, 456–459.

[bb30] Thromat, N., Noguera, C., Gautier, M., Jollet, F. & Duraud, J. P. (1991). *Phys. Rev. B*, **44**, 7904–7911. 10.1103/physrevb.44.7904 9998720

[bb31] Tim, K. T. & Olander, D. R. (1988). *J. Nucl. Mater.* **154**, 85–101.

[bb32] Tuilier, M. H., Dexpert-Ghys, J., Dexpert, H. & Lagarde, P. (1987). *J. Solid State Chem.* **69**, 153–161.

[bb33] Xu, Y., Yamazaki, M. & Villars, P. (2011). *Jpn. J. Appl. Phys.* **50**, 11RH02.

[bb34] Yabashi, M., Yamazaki, H., Tamasaku, K., Goto, S., Takeshita, K., Mochizuki, T., Yoneda, Y., Furukawa, Y. & Ishikawa, T. (1999). *Proc. SPIE*, **3773**, 2–13.

