# Supplementary material for: In situ XAFS–XRD study of the Zr–Y2O3 interaction at extra-high temperatures
Source: J Synchrotron Radiat. 2024 May 31;31(Pt 4):810–20. doi: 10.1107/S1600577524003321 (PMC11226155; doi:10.1107/S1600577524003321)
Supplement: Supplementary file 1 [file s-31-00810-sup1.pdf]

JOURNAL OF SYNCHROTRON RADIATION

Volume 31 (2024)

Supporting information for article:

In situ XAFS-XRD study of the Zr-Y2O3 interaction at extra-high temperatures

Ayumi Itoh, Satoru Matsuo, Kenta Yoshida, Kenji Konashi, Rikuto Ikuta, Keisuke Niino, Yuji Arita, Masaaki Kobata, Tatsuo Fukuda, Tohru Kobayashi, Hajime Tanida and Tsuyoshi Yaita

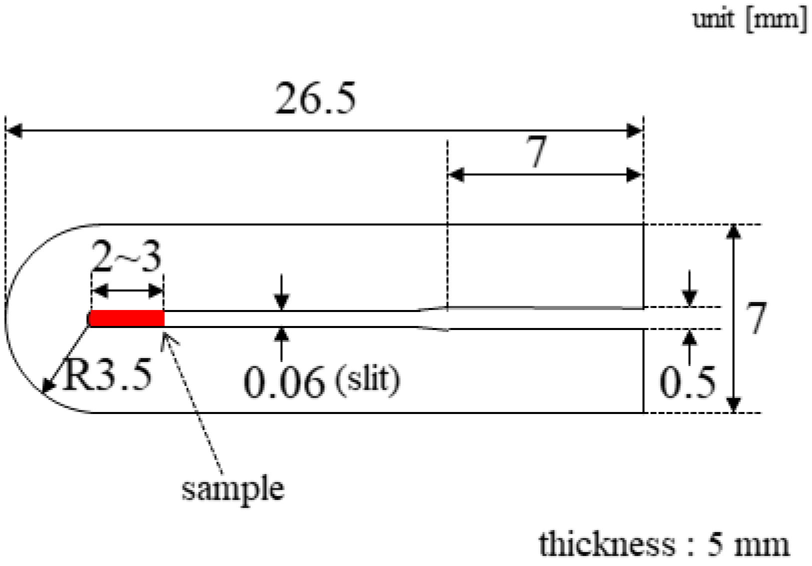

Figure S1 Detailed figure of the sample holder.

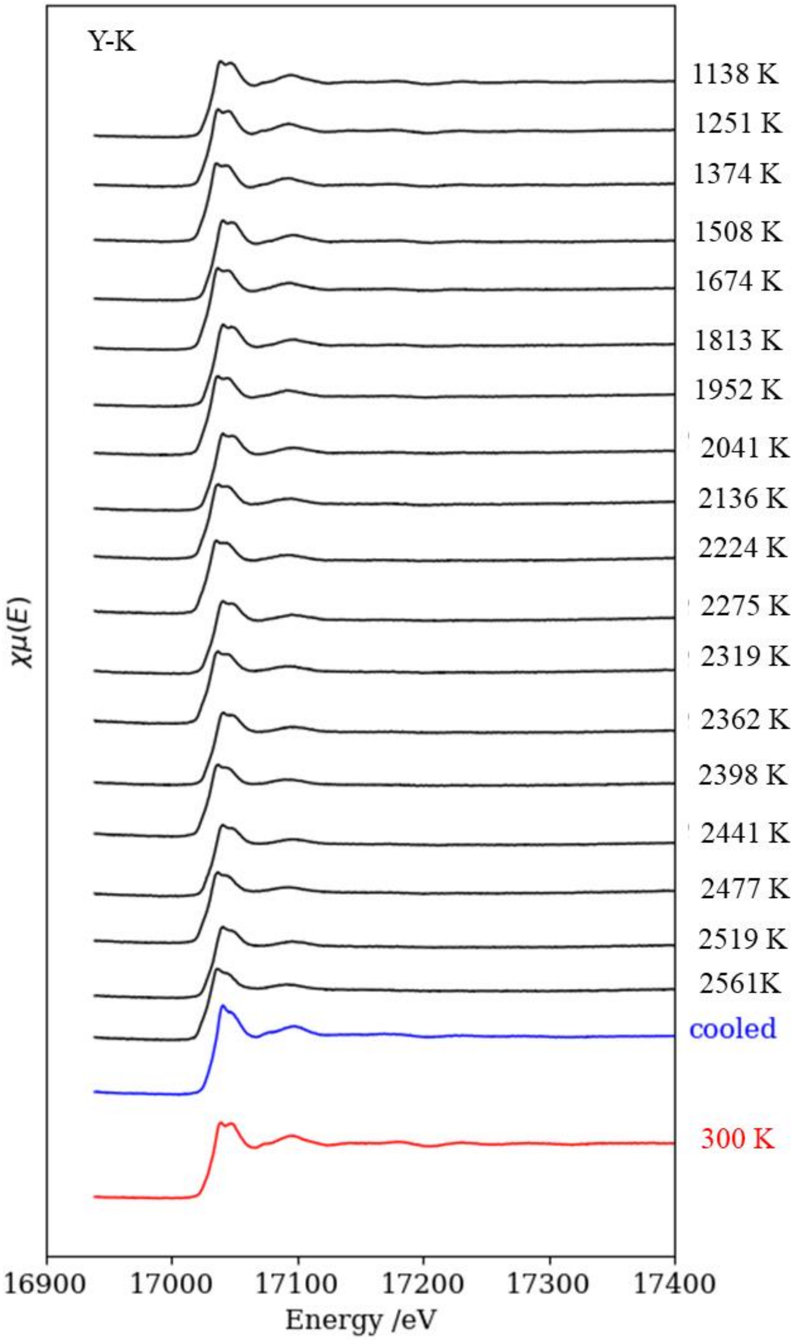

Figure S2 XAFS spectra at the Y-K edge.

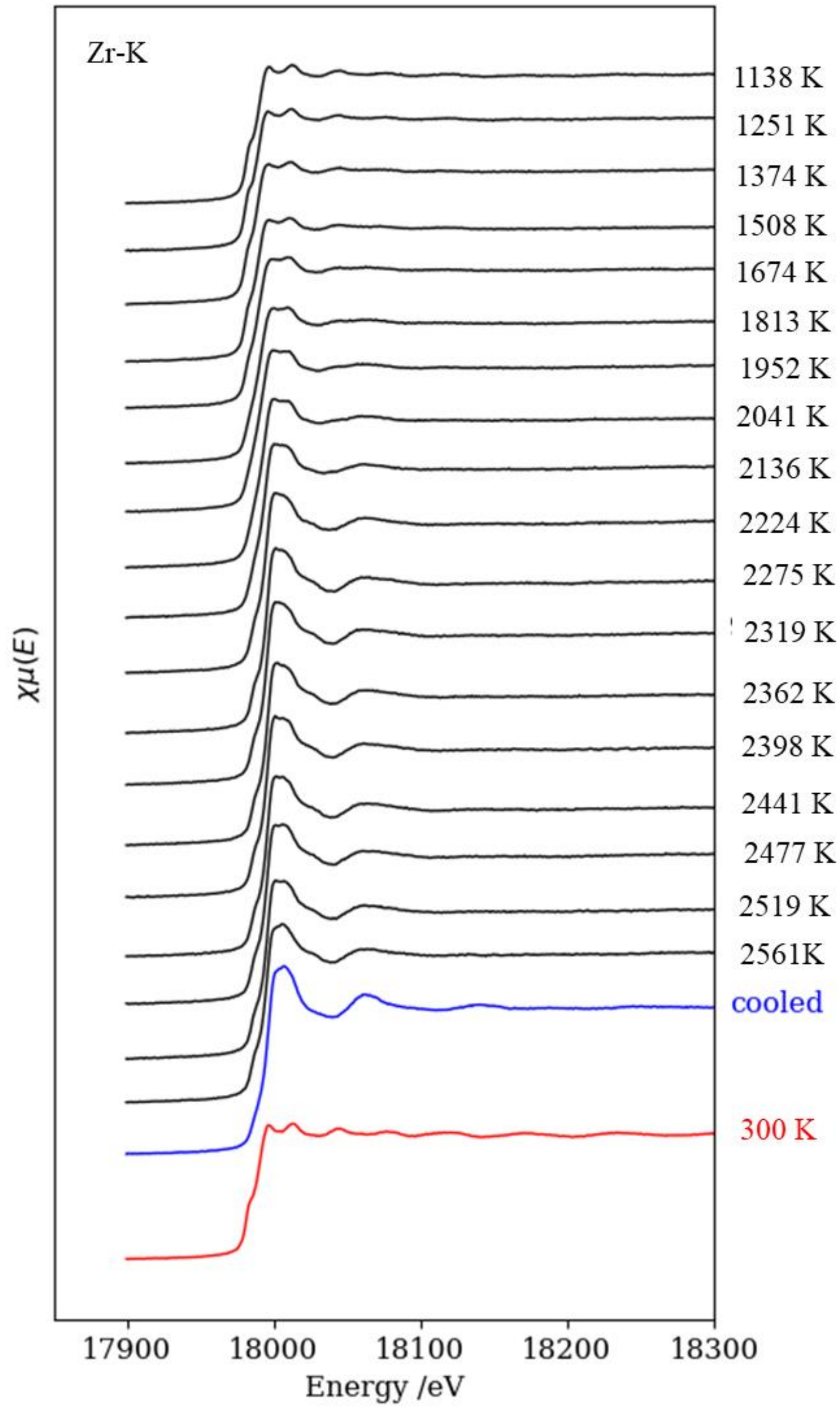

Figure S3 XAFS spectra at the Zr-K edges.
